# Biodegradable polymer nanocomposites for ligament/tendon tissue engineering

**DOI:** 10.1186/s12951-019-0556-1

**Published:** 2020-01-30

**Authors:** Magda Silva, Fernando N. Ferreira, Natália M. Alves, Maria C. Paiva

**Affiliations:** 1 3B’s Research Group, I3Bs–Research Institute on Biomaterials, Biodegradables and Biomimetics, University of Minho, Headquarters of the European Institute of Excellence on Tissue Engineering and Regenerative Medicine, AvePark‐Parque de Ciência e Tecnologia, Barco, 4805‐017 Guimarães, Portugal; 2 ICVS/3B’s, Associate PT Government Laboratory, Braga/Guimarães, Portugal; 3 Department of Polymer Engineering, Institute for Polymers and Composites/i3N, University of Minho, 4800-058 Guimarães, Portugal; 4 2C2T-Centre of Textile Science and Technology, University of Minho, 4800-058 Guimarães, Portugal

**Keywords:** Biodegradability, Nanocomposites, Tendon/ligament tissue engineering

## Abstract

Ligaments and tendons are fibrous tissues with poor vascularity and limited regeneration capacity. Currently, a ligament/tendon injury often require a surgical procedure using auto- or allografts that present some limitations. These inadequacies combined with the significant economic and health impact have prompted the development of tissue engineering approaches. Several natural and synthetic biodegradable polymers as well as composites, blends and hybrids based on such materials have been used to produce tendon and ligament scaffolds. Given the complex structure of native tissues, the production of fiber-based scaffolds has been the preferred option for tendon/ligament tissue engineering. Electrospinning and several textile methods such as twisting, braiding and knitting have been used to produce these scaffolds. This review focuses on the developments achieved in the preparation of tendon/ligament scaffolds based on different biodegradable polymers. Several examples are overviewed and their processing methodologies, as well as their biological and mechanical performances, are discussed.
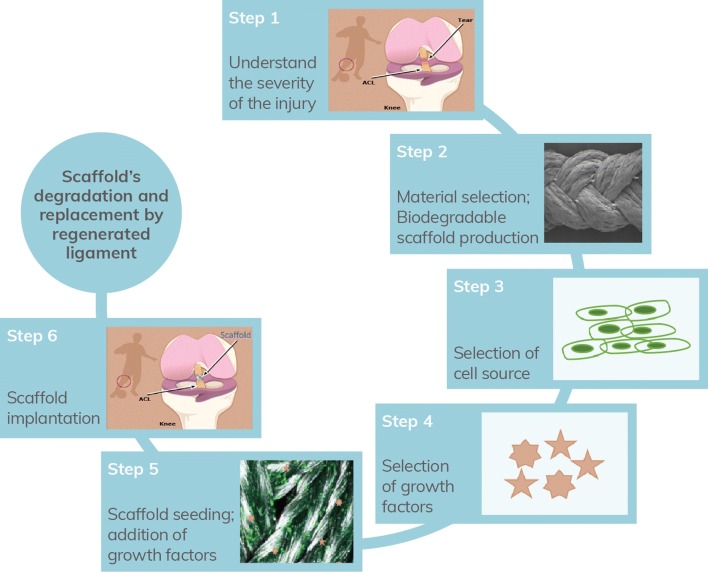

## Background

Tendons and ligaments have poor regeneration capacity with low cell density and low nutrient and oxygen requirements [1]. Injuries in these tissues such as in anterior cruciate ligament (ACL) are frequent in athletes and in elder and active working people, which cause joint instability accompanied by pain, disability, progressing of degenerative diseases and often, surgical interventions [2].

Current surgical reparative techniques rely on tissue replacement with auto- or allografts [3]. Despite excellent outcomes in terms of short-term results, serious complications are related to their usage and 5-year studies show that patients have instability and pain [4]. The main problems about the use of autografts include the need of additional surgery with potential donor harvest site infection and pain. On the other hand, concerns about using allografts are limited graft availability or even the risk of disease transmission, bacterial infection and the possibility of immunogenic response elicited in the host [5–7]. The need to address the shortcomings of existing strategies has prompted the investigation of synthetic and non-degradable substitutes.

The development of non-degradable synthetic ACL substitutes has emerged since the early 1970s and offer advantages over autograft or allograft [7]. They allowed a rapid rehabilitation, avoid donor tissue morbidity, and provide improved knee stability, not losing their strength during tissue revascularization [8, 9]. Thus, in 1973, Proplast, a combination of polyaramid fibers and ethylene polymers allowed cellular ingrowth and received Food and Drug Administration (FDA) approval for use as a ligament substitute [7]. Other commercial devices have emerged and have received the FDA approval as permanent prosthetic devices [8], such as a Gore-Tex device made with woven polytetrafluoroethylene fibers [8, 10] that was used between the mid 1980s and mid 1990s. In the early 1980s, the use of a polypropylene braid as a ligament augmentation device was proposed-Kennedy LAD device. Other devices were produced with polyester composites such as a polyester mesh in the case of the Leeds-Keio device. A second-generation of the Leeds-Keio device was made available in 2003. Distinct Polyethylene terephthalate devices were produced including Trevira-Hochfest, Proflex device, ProPivot and Ligament Augmentation and Reconstruction System (LARS) [10]. Initial enthusiasm for these devices was later faded by reports of complications and are not currently recommended for ACL repair [11]. They supply enough initial tensile strength, but all fail over time, with several limitations specific to their use: device creep, mechanical failure or mechanical mismatch with native tissue, problems with synovitis, chronic effusions, recurrent instability and early knee osteoarthritis [9–11]. Because of these complications, FDA has since removed these synthetic ACL grafts from the market. Thus, no synthetic replacements for ACL reconstruction are unconditionally approved for medical use in the United States [7]. The deficiencies of current approaches combined with the significant impact of these injuries on the community in terms of social, economic and health have prompted the research of tissue engineering (TE) approaches for tendon/ligament regeneration [2, 7, 9]. Thus, TE proposes alternative approaches combining cells with 3D scaffolds to mimic the mechanical and chemical cues of native extracellular matrix (ECM), and/or bioactive molecules to biochemically stimulate cells growth [9].

Specific cell types are incorporated into the scaffold which will be implanted into the host and interact with native cells and growth factors [12]. Interactions between cells and material’s scaffold are very important since materials could interfere with cells’ adhesion, proliferation and differentiation [8]. Ideally, cells should be readily available and have potential to proliferate and elaborate an ECM similar to native ligaments/tendons [13]. Resident tendon and ligament fibroblasts are the logical candidates for their regeneration. However, accessing them is difficult due to their intrasynovial location and exhibited a limited quantity and modest proliferative potential, which have restricted their usage. With the advancement of stem cell technology, pluripotent and multipotent stem cells for ligament/tendon tissue engineering have been more and more used [7] and include embryonic stem cells (ESCs), induced pluripotent stem cells (iPSC), bone marrow mesenchymal stem cells (BMSCs) and adipose-tissue-derived mesenchymal stem cells (AMSCs) [9].

The scaffold acts as a temporary engineered replacement of the native ECM with similar mechanical and functional characteristics [14, 15] and will gradually degrade, being slowly resorbed by the surrounding tissue, and replaced while a new natural tissue is resynthesized [3, 7, 12, 16].

The scaffold should mimic the properties of the native tissue, not only in terms of mechanical function [17], but also proper topography, geometry and porosity to recreate the native microenvironment and aid the cell adhesion, growth [14] and differentiation of the populating cells [17].

By labelling cells with quantum dots (QDs), it is possible to analyze variations in terms of number of cell populations adherent on different topographical regions, by counting cells labeled with QDs of the respective color. These QDs are readily incorporated by most cells’ lines and, at moderate concentrations and incubation times, do not cause acute cytotoxicity [18]. Besides, it has been found that the interaction between these nanoparticles and mesenchymal stem cells (MSCs) may influence their self-renewal, function and differentiation. Graphene-QDs, within a nontoxic concentration, promoted an osteogenic differentiation of MSCs, with gene activation and protein expression. Moreover, Graphene-QDs also promoted adipogenic differentiation of MSCs, which confirms that the pluripotency ability of MSCs was preserved [19].

Pore interconnectivity throughout an implant favors the distribution of nutrients, cell migration, metabolic waste removal and the tissue ingrowth, enhancing its regenerative properties [6, 20]. The long-term clinical success of scaffold also requires biocompatibility [6, 14] which is the ability of a material to perform with an appropriate host response in a desired application. It is not only dependent on the material characteristics but also on the situation in which the material is used and the toxicity of the degradation products [21, 22]. To improve biocompatibility and biofunctionality, extracellular matrix proteins and growth factors such as insulin like growth factor I (IGF-I), transforming growth factor-β (TGF-β) or basic fibroblast growth factor (bFGF) [7] have been incorporated into scaffolds to promote ligament/tendon regeneration [23].

Regarding the regulatory aspects of these TE scaffolds, they are generally under the category of medical devices. Medical devices are products or equipment generally intended for medical use. In European Union (EU), they are strictly regulated by both national competent authorities and by the European Medicines Agency (EMA). The adopted regulation in EU for such devices is Regulation (EU) 2017/745 on Medical Devices. In USA, the extensive regulatory requirements are defined by FDA. Moreover, when TE scaffolds are combined with cells, the classification of their category is not straightforward, depending on the cell type, and varying with the Regulatory Agency, e.g. FDA and EMA have distinct regulatory aspects. Also, their approval would be more complex: in fact, an acellular scaffold should face less regulatory scrutiny than approaches utilizing allogeneic or xenogeneic cells, iPSC, ESCs, or even significant ex vivo manipulation of autologous cells. The introduction of cells as a component in TE introduces attendant risks associated with possible immunogenicity, teratoma formation, cell culture adaptation/morphogenesis, or contamination which must be addressed to assure safety. In summary, the regulation of TE products is time-consuming, with an average time from pre-clinical/clinical studies to the market of about 15 years, and extremely high cost. There are already several papers/book chapters in the literature just devoted to the clinical translation of TE constructs and the associated regulatory aspects [24].

Despite the variety of TE solutions and biodegradable polymers proposed for ligament/tendon TE, they haven’t yet reached the clinic or even pre-clinics because they still exhibited problems related to the inadequacy of mechanical properties, degradation rate and biological response that are necessary to overcome [1]. For instance, there appears to be no consensus in the literature as to the nature of the scaffold material that is most suitable for clinical trials. So, further research is required to optimize tissue engineered ligament/tendon scaffolds before clinical application.

Thus, the selection of biodegradable and biocompatible materials with adequate degradation rate, structural and mechanical properties that mimic the organization of the ligaments/tendons represents a critical feature in the development of a successful scaffold.

## Biodegradable polymers for ligament/tendon tissue engineering

Biomaterials are natural or synthetic materials designed to interact with the biological systems, with an intended function in the body or to treat, augment or replace any tissue or organ [25, 26]. Successful scaffolds should be biocompatible and maintain the mechanical properties until it is replaced by native tissue, disintegrating into smaller fragments along the replacement process, being absorbed and excreted by the body [27]. Understanding the scaffold’s materials degradation behavior is very important when designing a new scaffold since it may alter its physicochemical properties and hence, its functionality or even its biological response [28]. Thus, the scaffolds’ biocompatibility is intimately related to the scaffolds’ composition, which should not cause any significant systemic inflammation or local reaction [29], but also to its biodegradation, since the degradation products should be nontoxic and metabolized by the body [17, 29]. Scaffold’s polymer degradation rate plays an important role in the cellular vitality and growth and should be similar to the rate of new tissue formation, allowing the occupation of the scaffolds’ space by the new tissue formed [29].

When in contact with surrounding fluids, polymers degrade by chain scission yielding low molecular weight species, oligomers and monomers [30]. All biodegradable polymers contain hydrolysable bonds making them prone to chemical degradation via hydrolysis or enzyme-catalyzed hydrolysis [29, 31, 32]. Synthetic polymers, in contrast to natural polymers, are less susceptible to enzymatic hydrolysis, and so tend to degrade by simple hydrolysis [33]. As a consequence of the water soluble degradation products (chemical phenomena), erosion of the material can occur (physical phenomena) [29].

Several natural polymers such as collagen (Col), silk, chitosan (CHI), hyaluronic acid (HA) and synthetic biodegradable polymers such as polylactic acid (PLA), polyglycolic acid (PGA), poly(lactic-co-glycolic acid (PLGA), poly(ε-caprolactone) (PCL), as well as biodegradable based polymeric composites have been used to produce scaffolds for tendon and ligament TE [1, 3, 5, 14, 34], in the form of gels, membranes, or three dimensional (3D) fibrous scaffolds.

PLA, PGA and PLGA are considered biocompatible, causing just minimal or mild foreign body reaction, since their hydrolytic degradation products (lactic and glycolic acids) are normally present in the metabolic pathways of the human body [30]. However, their bulk degradation may occasionally lead to local inflammation due to accumulation of acidic degradation products that cannot be easily disposed. PCL is also biocompatible and degrades at a much lower rate than PLA, PGA, and PLGA, making it attractive for long-term scaffolds such as tendon/ligament scaffolds [30]. For instance, ACL regeneration and subsequent functionality usually requires at least 6 months [35]. For such applications materials with a slower degradation should be selected [33, 35].

The polymer degradation rate is strongly influenced by several parameters such as the morphology, molecular weight and its distribution, crystallinity degree, glass transition temperature and environmental conditions (medium, temperature, and pH) [36]. It can be controlled by varying composition, molecular weight, processing conditions or even blending with biodegradable polymers with different characteristics [12, 33]. For example, several degradation profiles and mechanical properties are possible to obtain just by using different fibers, composed of materials with different degradation rates, and varying their diameter or architecture [33].

Most degradation experiments are performed in vitro by incubating the scaffold in phosphate buffered saline (PBS) at body temperature (37 °C). However, in vivo degradation is significantly different and occurs faster than in vitro degradation due to the tissue response. Once implanted, the scaffold is identified as a foreign body creating an inflammatory response. This induces the migration of leucocytes and macrophages to the implant site, forming reactive products as hydrogen peroxide that oxidize the polymer. The degradation products will be removed from the implantation site by the lymphatic system and subsequently secreted from the body. The in vivo mass loss can be also increased by mechanical stimulations and cellular activity. Besides, the size and the shape of the scaffold influence its degradation rate. Larger implants require longer degradation times [30].

Most of the research for tendon tissue regeneration proposes the use of Col alone or mixed with other molecules, such as proteoglycans, to produce scaffolds in form of sponges, aligned extruded Col fibers or electrochemically-aligned Col [37]. Regarding ligament regeneration and specifically tissue-engineered ACLs, Col and the L enantiomer of PLA, Poly (L-lactic) acid (PLLA), have been the most used materials to produce biodegradable scaffolds, although some of them do not achieve more than 20% of the ultimate tensile strength of native ACL [8]. PLLA has demonstrated reasonable properties in terms of material strength and resorption rate [38], as well as it does not cause a permanent foreign body reaction [11].

All the referred biodegradable polymers can be easily processed into fibers and fibrous scaffolds. However, each of these polymers has exhibited some inadequacies for tendon/ligament applications, such as inadequate mechanical properties and degradation rate [30]. Also, despite the variety of approaches on ligament tissue-engineering, only a few of them were tested in vivo, using dogs, rabbits, goats and sheep [8].

### Natural polymers

Natural polymers such as Col, alginate (ALG), CHI, HA, silk, fibrin and cellulose are attractive materials for biomedical applications due to their biocompatibility and capacity to structurally mimic the native ECM [39]. These polymers are capable of hydrolytic or enzymatic degradation [12], since they have a similar composition to macromolecular substances which are recognized by the biological environment and metabolized [40]. For that reason, the common problems caused by synthetic polymers are frequently avoided, such as stimulation of chronic immunological reactions and toxicity, as well as lack of cell recognition [25]. Natural polymers contain functional groups that allow a chemical conjugation with other molecules, such as growth factors [12, 41]. This feature may be beneficial for their further application in tendon/ligament scaffolds, as described in Table 1.

**Table 1:** Natural biodegradable polymers commonly used in tendon/ligament regeneration

Natural biomaterial	Advantages	Disadvantages
Collagen	Biocompatible; major component of ligaments [7]; reasonable mechanical properties [9]	Poor mechanical strength; [7] risk of immunogenicity; [27] fast degradation [42]
Silk	Good mechanical properties; slow rate biodegradation [9]; loses its strength after 1 year, in vivo [33]	Limited cell adhesion [7]
Alginate	Biocompatible ECM component; can be in sponge or hydrogel form [7]; proper substrate for fibroblasts growth and collagen type I production [9]	Lacks mechanical properties [7]
Hyaluronic acid	Biocompatible; can be in sponge or hydrogel form [7]	Natural form with very short degradation time [43]
Chitosan	Biocompatible; can be in sponge or hydrogel form [7]; proper substrate for fibroblasts growth and Col type I production [9]	Lacks mechanical properties [7]

In spite of various advantages, natural polymers typically have relatively poor mechanical properties [44] and present low processing ability when compared to the synthetic ones, which limit their application [17, 25]. Besides, these polymers often suffer batch-to-batch variability in molecular weight and purity, which represent low reproducibility amongst different samples of the same material [12, 17].

#### Collagen

The most obvious and common choice for ligament and tendon TE is Col type I because of its prevalence in the native tissues [3, 45–48]. It forms the connective tissue on which the fibroblasts adhere and proliferate [1, 39]. For that reason, Col was the first natural scaffold’s material to be used in ligament reconstruction [1]. Purified Col derived from animal tissue requires crosslinking to remove foreign antigen, avoid potential disease transmission, improve its mechanical strength and slow down its degradation rate [33]. However, even after physical or chemical crosslinking of Col, the collagenous scaffolds fail to reproduce the mechanical properties of native collagenous tissues, the support of mechanical loading decreases over time [13, 14] and suffer relatively fast in vivo degradation [33].

Dunn et al. [46] extruded Col fibers and crosslinked them to produce collagen fibrous scaffolds. Rabbit ACL and patellar tendon (PT) fibroblasts were seeded onto Col scaffolds and adherence and viability in vitro was found in both cases [46]. Bellincampi et al. [47] determined the in vivo fate of autogenous ACL and skin fibroblasts-seeded onto collagenous scaffold as a function of fibroblast source, implantation site and time. The cultured cells were seeded onto Col fiber scaffolds and implanted in rabbits. The seeded skin and ACL fibroblasts survived for at least 4–6 weeks after implantation and the fibroblast type seemed to have no influence on the viability. However, they verified a complete resorption of the scaffolds after 6 weeks [47].

Concerns about the Col mechanical performance, immunogenicity and leaching of chemical crosslinking agents have led to explore alternative scaffold materials [7, 13], such as silk, polysaccharides or synthetic polymers. Nevertheless, new crosslinking strategies as well as scaffolds with a braid-twist design [7] or even decellularized ECM-derived Col scaffolds [14] are still being explored to achieve Col scaffolds with more favorable properties for ligament regeneration. Walters et al. [48] have recently developed Col type I fiber-based scaffolds for ACL ligament with a braid-twist design and evaluated the effect of crosslinking method and the addition of gelatin on the mechanical properties. Although the crosslinked scaffolds without gelatin exhibit lower ultimate tensile strength (UTS) than native human ACL but with a similar Young’s Modulus, improvements are still desired [48]. According to Noyes and Grood [49], ACLs from younger human donors (16–26 years of age) exhibited a UTS of 37.8 ± 9.3 MPa and a Young’s modulus of 111 ± 26 MPa [48].

#### Silk

Like Col, silk has been effectively used in ligament regeneration approaches [2, 16, 50–56] being easily fabricated into gels, films and braided or knitted fibers [3].

Its main advantage is its remarkable tensile strength and toughness compared to most natural materials although being lower than native human ACL [1]. Silk fibers lose their tensile strength in 1 year and undergo complete proteolytic degradation within 2 years in vivo [7]. This allows a gradual transfer of mechanical load from the scaffold to the neoligament [57].

In addition, silk biomaterials are biocompatible in vitro and in vivo [58]. Silk scaffolds have supported attachment and proliferation of several primary cells and cell lines [58], such as human BMSCs and fibroblast [55] as well as synthesis of fibroblastic markers with the application of mechanical stimulation [7, 16].

**Fig. 1:**
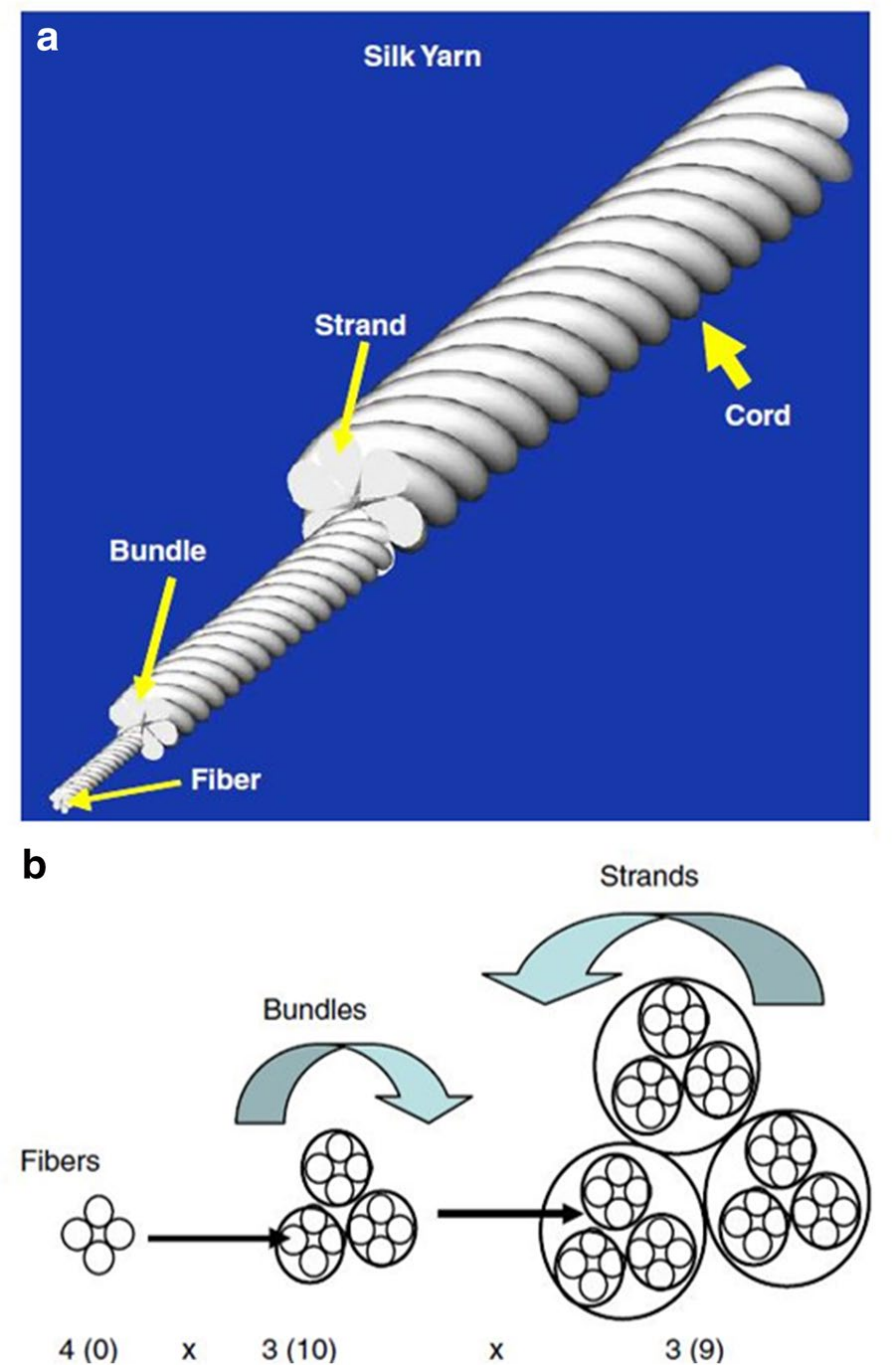
**a** and **b** Structure of a twisted or cable yarn. Fibers are combined to form bundles, bundles to form strands, and strands to form cords. Yarns were labeled: A(a) × B(b) × C(c), where A, B, C represent the number of fibers/bundles/strands in the final structure, respectively and a, b, c is the number of turns per inch on each of the hierarchical levels (Adapted from [52])

**Fig. 2:**
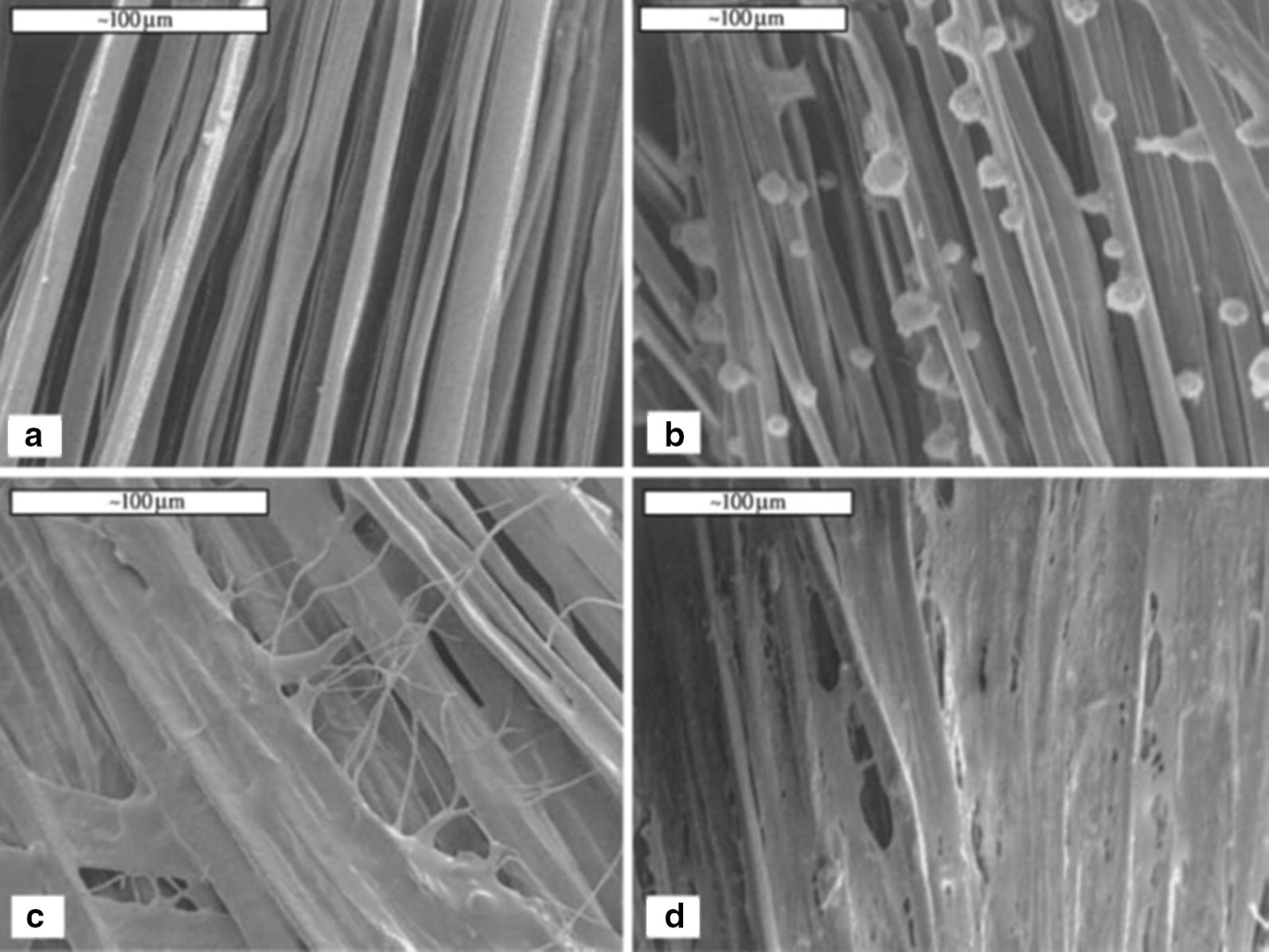
Scanning electron microscopy (SEM) showing adherence, proliferation and cell sheet formation by human BMSCs on the silk cord matrix prior to seeding (**a**), time 0 following seeding (**b**), 1 day (**c**), and 14 days (**d**). Scale bars = 100 mm (Reprinted with permission from [16])

Recently, Teuschl et al. [53] reported the braiding of silk fibers into wire rope–like structures to produce scaffolds that were boiled in borate buffer to remove sericin. The resulting silk ACL grafts were seeded with autologous stem cells and were able to stimulate ACL regeneration under in vivo conditions, using mountain sheep models. The seeded scaffolds exhibited UTS and elasticity values comparable to native ovine ACL [53]. Several textile methods such as twisting or cabling have been used to design TE scaffolds—see Fig. 1 [52]. Similarly, Chen et al. [55] and Altman et al. [16] showed that silk fibroin, is nonantigenic, biocompatible, and allow the BMSCs attachment, proliferation and differentiation toward ligament lineage Fig. 2.

In their study, Chen et al. [55] studied wire-rope silk matrices and silk films, modified with a short polypeptide. Modified silk matrices improved human BMSCs and ACL fibroblasts adhesion and showed higher cell density and Col production, over 14 days in culture when compared with the non -modified matrices [55]. The 6-cord silk wire-rope scaffold produced by Altman et al. [16] not only supported the aforementioned attachment, expansion and differentiation of BMSCs but also presented slow degradability and mechanical properties similar to those of the native human ACL [16]. Fan et al. [50] prepared a scaffold by rolling a knitted microporous silk mesh around a braided silk cord. MSCs seeded on these scaffolds [50] proliferated and differentiated into fibroblast-like cells by expressing collagen I, collagen III and tenascin-C genes in mRNA level. MSCs seeded scaffolds were implanted in a pig to regenerate the ACL. A remarkable scaffold degradation was observed, but the maximum tensile load of regenerated ligament was be maintained after 24 weeks of implantation. The tensile loss caused by the degradation of scaffold was compensated by the new tissue formed. MSCs showed robust proliferation and fibroblast differentiation, at 24 weeks postoperatively [50].

**Fig. 3:**
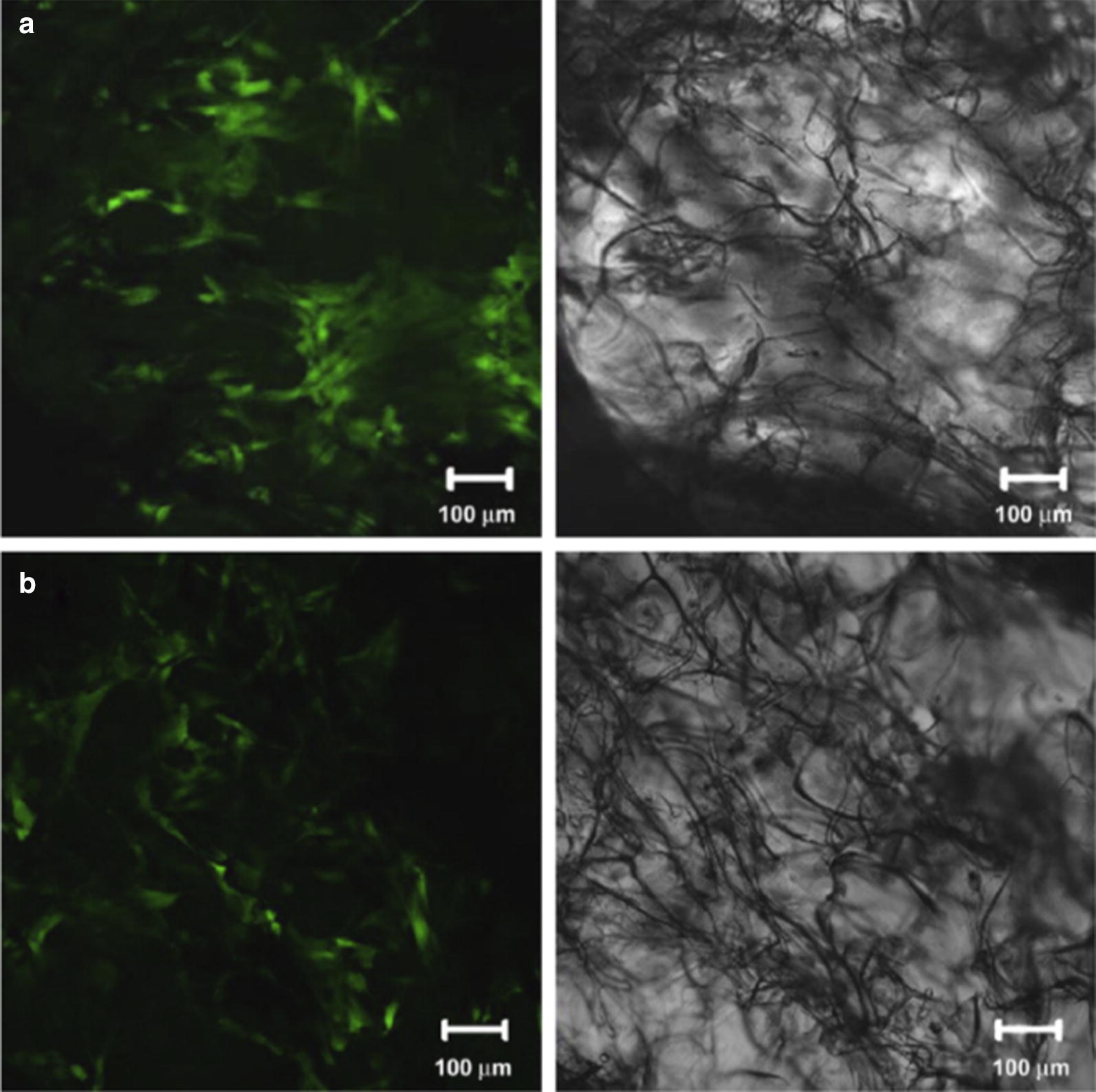
Fluorescence images of implants with silk scaffolds-with BMSCs (**a**) and ACL fibroblasts (**b**) at 4 weeks post implantation. Scale bars = 100 mm (Reprinted with permission from [56])

Liu et al. [56] proposed a combined scaffold that incorporates microporous silk sponges into a knitted silk scaffold for ACL tissue engineering. BMSCs and ACL fibroblasts were seeded onto the scaffolds and cultured in vitro for 2 weeks. To evaluate the in vivo survivability, BMSCs or ACL fibroblasts seeded on each silk scaffold and implanted in rabbits were examined at 4 weeks post implantation. BMSCs presented advantages over ACL fibroblasts, in terms of cell proliferation, glycosaminoglycan excretion, gene and protein expression for ligament-related ECM markers, and in vivo viability Fig. 3 [56].

#### Polysaccharides

HA fibers are another natural-origin alternative for ACL replacement [43, 59]. HA is an anionic polysaccharide naturally present in all soft tissues, being responsible for the maintenance of the normal extracellular matrix structure [43]. It is not immunogenic [43], being the main component of glycosaminoglycans, known for stimulating various in vitro tissue regenerative processes. The natural form of HA is in gel and has a very short degradation time. For that reason, some chemical modifications have been proposed to improve its processability and biodegradation [43]. The biological effects of HA, such as the improvement of cellular adhesion and proliferation as well as anti-inflammatory character, could enhance ligament tissue regeneration [60]. For example, Cristino et al. [43] seeded MSCs into the HA-based prototype ligament scaffold, and verified that MSCs cells completely wrapped the scaffold fibers and expressed CD44, a receptor important for scaffold interaction, and typical ligamentous markers, such as collagen type I, type III, fibronectin, laminin, and actin [43].

CHI is a cationic polysaccharide with excellent adhesive properties and biocompatibility which has led to its application as a scaffold material in the field of musculoskeletal tissue engineering [60]. Due to their opposite charges, HA and ALG are usually combined with CHI to form polyionic complexes effective for scaffolds and with excellent adhesive properties [60, 61].

Table 2 summarizes the main studies that have used natural biodegradable polymers for ligament/tendon tissue engineering and highlights the major outcomes for the proposed scaffolds in terms of mechanical and in vitro/in vivo properties.

**Table 2:** Performance of natural biodegradable polymers in ligament/tendon TE

Material	Scaffold	Application	In vivo*/*in vitro	Mechanical response				Biological response	Refs.
				Young’s modulus (MPa)	Stiffness (N/mm)	Max. strength (MPa)	Max. load^a^ (N)		
Collagen	Aligned parallel 200 extruded-crosslinked col fibers; coating with Col	Ligament	In vitro (ACL and PT fibroblasts)	ND		ND		Cells adhesion, proliferation ACL cells: ovoid shape; no alignment; PT cells: elongation	[46]
	Aligned parallel 200 extruded-crosslinked Col fibers		In vivo, rabbit model	ND		ND		Skin/ACL fibroblasts survived 4-6 weeks, after implantation; scaffold resorption after 8 weeks	[47]
	(i) Extruded-crosslinked Col fibers from bovine Achilles tendon (ii) Extruded-crosslinked Col fibers from rat tail tendon (iii) Fiber-embedded gel scaffolds		In vitro (fibroblasts)	(i) 359.6± 28.4 (ii) 995.1 ± 144 (iii) 83.4 ± 10.8	ND	(i) 36.0 ± 5.40 (ii) 106.1 ± 13.90 (iii) 5.4 ± 0.4	ND	Non-uniform cells distribution	[45]
	Braid-twist scaffold of extruded-crosslinked Col fiber i) with or ii) without gelatin		In vitro (primary rat ligament fibroblasts)	(i) 6.32 ± 0.95 (ii) 148 ± 170	ND	(i) 1.07 ± 0.06 (ii) 19.3 ± 3.10	ND	Cell adhesion, proliferation; only (ii) exhibited increased cellular activity after 21 days of culture	[48]
	Woven scaffold by electrochemically aligned Col threads (yarns)	Tendon/tendon	In vitro (MSCs)	~ 520	ND	~ 65	ND	MSCs adhesion, proliferation, elongation after 35 days in culture Tendon-specific/related markers	[62]
	Sponges (L/W/T = 23/9/3 mm) i) Non-stimulated scaffolds ii) Mechanically stimulated scaffolds		In vitro (rabbit MSCs)/In vivo, rabbit model	(i) 343.2 ± 21.2 (ii) 441.2 ± 26.3	ND	(i) 50.2 ± 9.2 (ii) 72.1 ± 11.1	(i) 271.5 ± 17.5 (ii) 339 ± 11.4	MSCs adhesion and alignment (i) and (ii) excellent cellular alignment, after 12 weeks of culture Tendon-related ECM components	[63]
Silk	Knitted microporous silk mesh rolled up around a braided silk cord	Ligament/ligament	In vitro (MSCs)/in vivo, pig model	ND	58.5 ± 17	ND	398 ± 70	MSCs adhesion, proliferation, differentiation Ligament-specific ECM markers at 24 weeks post-operatively	[50]
	Wire rope–like scaffolds (i) Cell seeded-scaffold (ii) Non-seeded scaffold		In vivo (autologous stem cells), sheep model	ND	194 ± 27	ND	1450 ± 65	(i) Higher cell content in the inner part, after 6 months (i), (ii) Silk fiber degradation after 12 months	[53]
	(i) Wire rope of multifiber (ii) Parallel multifiber (theoretical)		In vitro (BMSCs)	ND	(i) 354 ± 26 (ii) 1740	ND	(i) 2337 ± 72 (ii) 2214	(i) BMSCs adhesion, proliferation, differentiation Ligament-specific markers, after 14 days of culture (ii) ND	[16]
	Knitted silk mesh integrated: (i) Aligned electrospun fibers (ii) Random electrospun fibers (iii) Without fibers		In vitro (MSCs)	ND	(i) 22.12 ± 1.2 (ii) 19.21 ± 0.9 (iii) 16.24 ± 0.8	ND	(i) 129 ± 7.4 (ii) 106 ± 6.2 (iii) 93.2 ± 5.6	(i) MSCs proliferation, elongation, orientation along the fibers; ligament-related proteins increased when compared to (ii) scaffolds	[51]
	Microporous silk sponges incorporated into a knitted silk scaffold		In vitro (BMSCs and ACL fibroblasts)/in vivo, rabbit model	ND		ND		BMSCs with higher proliferation, ligament-related ECM markers and in vivo viability, comparing to ACL fibroblasts	[56]
			In vitro (MSCs)	ND	50 ± 4 (after 14 days of cell culture)	ND	257 ± 7 (after 14 days of cell culture)	MSCs adhesion, proliferation Ligament-specific ECM markers, after 15 days of culture	[64]
	(i) Wired (ii) Braided		In vitro (hFF^b^)	ND	(i) ~ 280 (ii) ~ 240 (wet)	ND	(i) ~ 1560 (ii) ~ 1610 (wet)	Inconclusive cell invasion, proliferation	[65]
(i) Silk (ii) PBS	Extruded fibers in knitted scaffolds (weft knitting)	Tendon/ligament	In vitro (L929 fibroblasts)	(i) 31.6 (ii) 7.9	ND	(i) 17.4 (ii) 8.2	ND	(i), (ii): L929 adhesion, proliferation (i) Differentiation, after 14 days of culture; (ii) cells with rounder shape	[66]
HA (HYAFF 11^®^)	Multilayered knitted cylindrical array of fibers	Ligament	In vitro (MSCs)	ND		ND		MSCs adhesion, proliferation, differentiation Ligament-specific ECM markers	[43]

^a^maximum tensile load (max. load); ^b^human foreskin fibroblasts (hFF)

### Synthetic polymers

Owing to their availability, ease of processability and reproducibility, synthetic polymers have been widely used to produce tendon/ligament scaffolds [14, 17]. Contrasting to the natural ones, synthetic polymers present low immunogenicity potential and are more versatile, enabling tailoring and controlling the chemical and physical properties [2].

Polyesters such as PCL and PGA, PLLA, poly(L-lactide-co-ε-caprolactone) (PLCL) and PLGA have been effectively used to produce mechanically strong and biodegradable scaffolds for tendon/ligament applications-Table 3 [14, 35]. These polymers are well characterized and have been approved by the FDA for certain human uses [37]. However, one of the disadvantages of synthetic polymers is the lack of biological cues for promoting cell adhesion and proliferation, which has to be overcome by, for example, applying a specific coating [37].

**Table 3:** Synthetic biodegradable polymers commonly used in tendon/ligament regeneration

Synthetic Biomaterial	Advantages	Disadvantages
PLLA	Slow degradation rate (10 months to 4 years) [33], better cell adhesion than PGA or PLGA. Easily manufactured [7]	Acidic degradation [7]
PCL	Easily manufactured; FDA approved material [7]; (over 3 years in vivo) [67]	Very slow degradation rate [7]
PGA	Easily manufactured; FDA approved material [7]	Rapid (6–12 months) [68] and acidic degradation [7]; lack of signaling molecules [61]
PLGA	Half-life of 1.5 months [68]; Degradation rate can be tailored by changing the ratio of PLA:PGA. Easily manufactured [7]	Acidic degradation [7]
PLCL	Properties can be tailored by changing the ratio of PLA:PCL. Good biocompatibility and mechanical properties; easily manufactured [69]	Excessively elastic for tendon regeneration [69]

#### Poly-α-hydroxyesters

PLGA is a linear aliphatic polyester that contains lactide and glycolide as its monomers [39]. It has been considered an attractive choice for ligament/tendon regeneration mainly due to its design flexibility and complete in vivo bioresorption [6, 14, 70–72]. Moffat et al. [70] produced a PLGA nanofiber-based scaffold for rotator cuff tendon tissue engineering. The influence of design in the attachment, alignment and gene expression of human rotator cuff fibroblasts on aligned and unaligned PLGA nanofiber scaffolds was evaluated. Aligned nanofiber scaffolds presented significantly better mechanical properties than those of the unaligned. The tensile modulus of the unaligned and aligned scaffolds averaged 107 MPa and 341 MPa, respectively, with mean ultimate tensile strength ranging from 3.7 to 12.0 MPa. The human rotator cuff fibroblasts exhibited a phenotypic morphology and attached preferentially along the nanofiber axis of the aligned scaffolds, whereas only random cell attachment was observed on the unaligned scaffold [70].

**Fig. 4:**
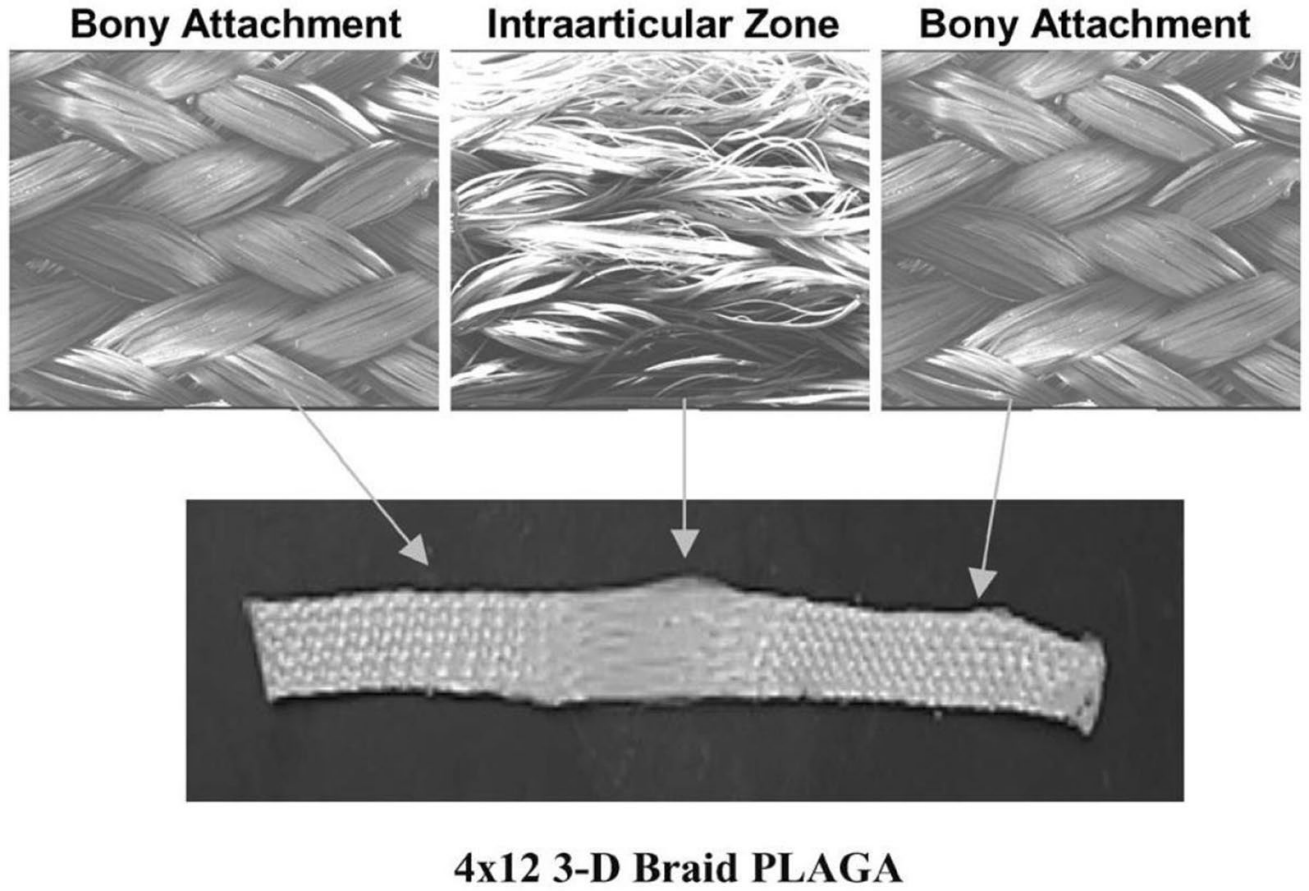
General configuration of ligament scaffold design for 3-D rectangular braid, with 3 regions: femoral tunnel attachment site, ligament region, and tibial tunnel attachment site (Reprinted with permission from [6])

**Fig. 5:**
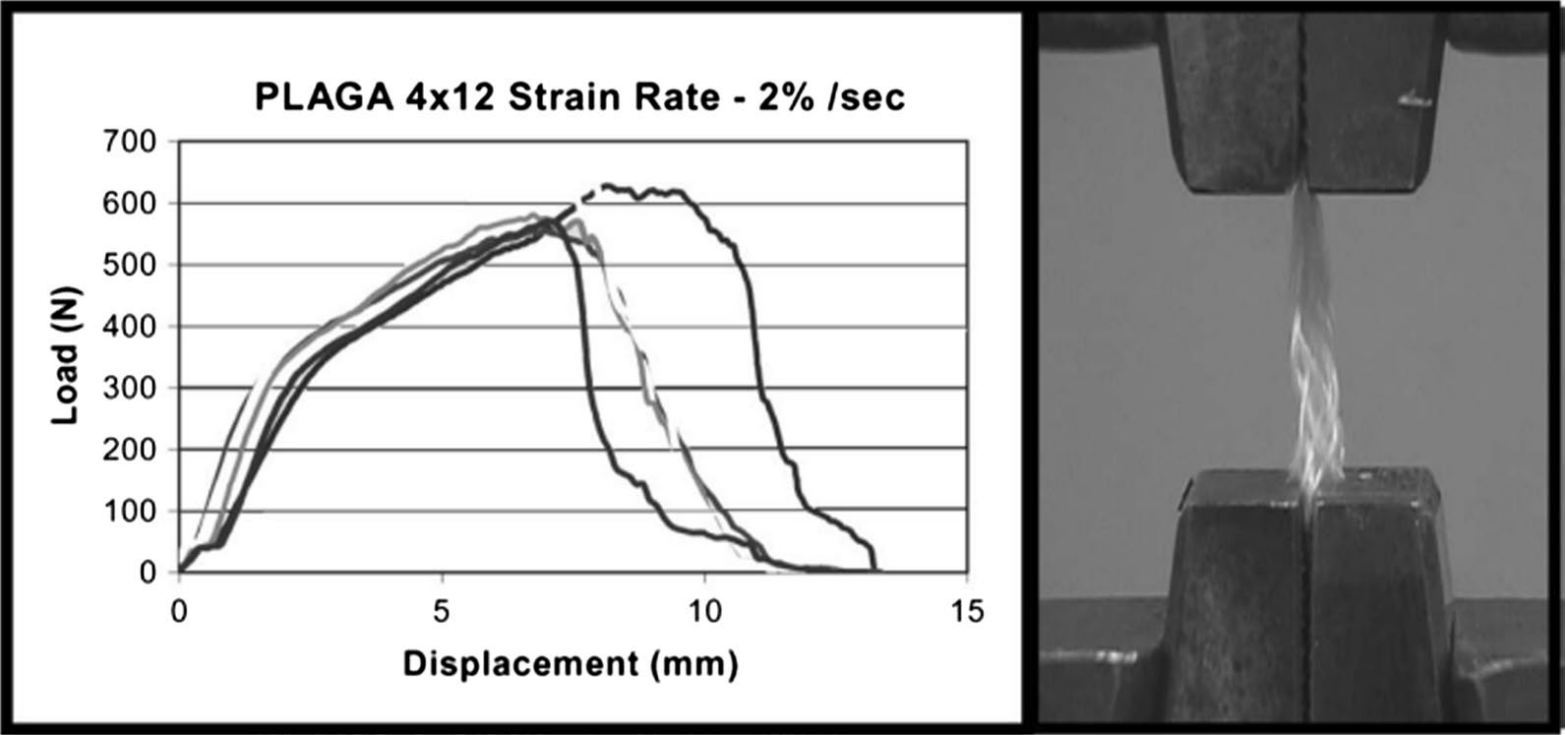
Load-deformation curve and photomicrograph of mechanical failure of the 4 × 12 PLGA 3-D rectangular braids at a strain rate of 2%/s (Reprinted with permission from [6])

Cooper et al. [6] proposed 3D braided scaffolds based on PLGA fibers, using a 3D circular braiding system and a rectangular braiding system for comparison. The 3D circular fibrous scaffold has the highest tensile loads of 907 ± 132 N, which was greater than the level for normal human physical activity. The stress–strain profile was found to be similar to that of natural ligament tissue. The scaffold porosity (175–233 mm) was adequate for tissue ingrowth. An example of the scaffold design for 3-D rectangular braid and the corresponding load–deformation curves of the 3-D rectangular braids is shown in Figs. 4 and 5, respectively. Primary rabbit ACL cells and BALB/C mouse fibroblasts adhered and spread on scaffolds. Both types of cells grew on the rectangular braided scaffold but only the ACL cells grew on the 3-D circular braids [6].

Braided and knitted scaffolds often require a gel system for cell seeding. In order to overcome this limitation, Sahoo et al. [71] proposed a biodegradable scaffold produced by electrospinning PLGA nanofibers onto a knitted PLGA scaffold. BMSCs were seeded on these scaffolds and on knitted PLGA scaffolds by immobilizing in fibrin gel. BMSCs produced abundant ECM with a higher expression of collagen-I, decorin, and biglycan on the scaffold with nanofibers demonstrating their potential to differentiate into tendon/ligament tissue.

The biodegradation of PLGA occurs mainly via chemical hydrolysis of the hydrolytically unstable ester bonds into lactic acid and glycolic acid, which are non-toxic and removed from the body by normal metabolic pathways [36]. However, its biodegradation occurs within weeks, which results in complete loss of mechanical strength and compromise the integrity of PLGA-based scaffolds throughout the ligament healing period that generally extends to months [14, 73]. For that reason, PLGA is usually combined with other polymers, such as PLA [35].

Regarding PLA, it is a linear aliphatic polyester, an homopolymer containing only lactide subunits as monomer [39]. It has a slow degradation rate [33] being widely suggested for several tendon-ligament scaffolds [11, 74–77]. It undergoes hydrolytic scission into lactic acid and is eliminated from the body mainly through respiration by the lungs, as CO_2_ [78]. This degradation occurs within a period between 10 months to 4 years depending on its molecular weight, crystallinity, shape and site of the implant [79].

**Fig. 6:**
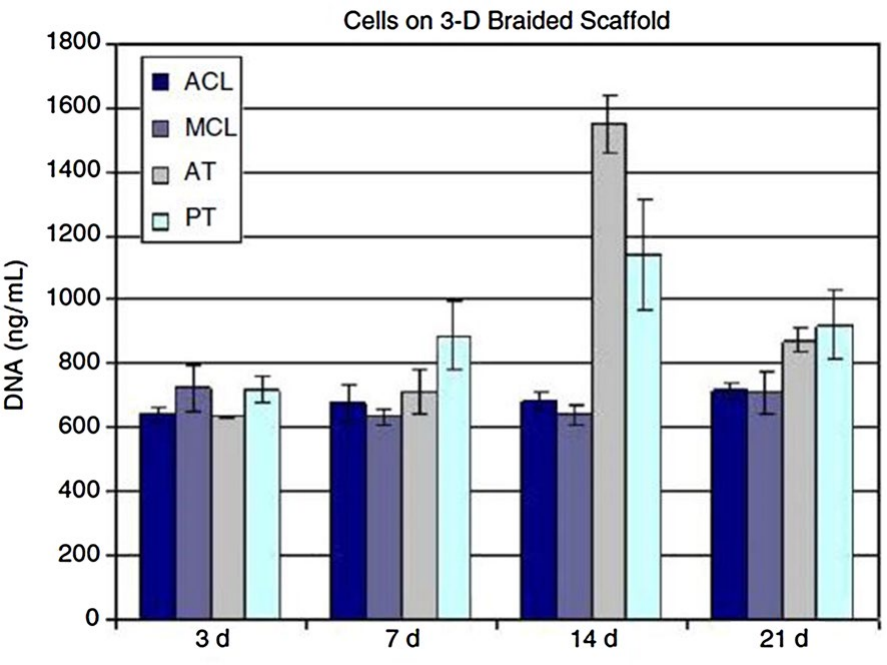
The cellular proliferation after culturing for 3, 7, 14 and 21 days on 5 × 5 PLLA 3D square braided scaffolds. The temporal cell growth of the ligament cells was slower as compared to the tendon cells [74]

Cooper et al. [74] cultured, in vitro, different types of cells derived from the ACL, medial collateral ligament (MCL), Achilles tendon (AT), and PT of rabbits on 3D braided PLLA scaffolds. This study revealed that all the primary connective tissue fibroblasts expressed genes associated with ligament differentiation but only PT and AT cells had the greatest in vitro proliferation on 3D braided scaffolds-Fig. 6. The 3D braiding geometry affected the matrix production of ACL cells, favoring the production of a filamentous matrix [74]. Lu et al. [72] and Laurencin et al. [11] reported an affinity of ACL fibroblasts to PLLA scaffolds. According to Cooper et al. [6] PLGA scaffolds produced by a circular braiding achieved higher tensile loads. For that reason, Lu et al. [72] also developed 3D braided PLLA scaffolds in a circular system. They verified that ACL fibroblasts conformed to the geometry of these PLLA scaffolds, being the cell attachment and proliferation increased when the scaffolds were coated with fibronectin (Fn). Fn is an important protein which is upregulated during ligament healing [72].

Concerning PCL, it is a hydrophobic polyester with semi-crystalline structure, containing caprolactone subunits [39]. It exhibits favorable biocompatibility, adequate mechanical strength, high elasticity as well as long degradation time which has prompted its application in tissue engineering [80]. Comparing to PLLA, PCL presents a slower degradation rate. However, its hydrophobicity may results in poor cell attachment and proliferation [81]. For that reason, when aiming tendon/ligament regeneration, PCL and derivatives are usually combined with other polymers such as CHI [82, 83], or simply coated with Col [80, 84]. In a study, electrospun PCL fibers were implanted in a rodent model for wound healing, showing evidences that PCL is nonimmunogenic, being integrated into local tissue without adverse reactions [85].

**Fig. 7:**
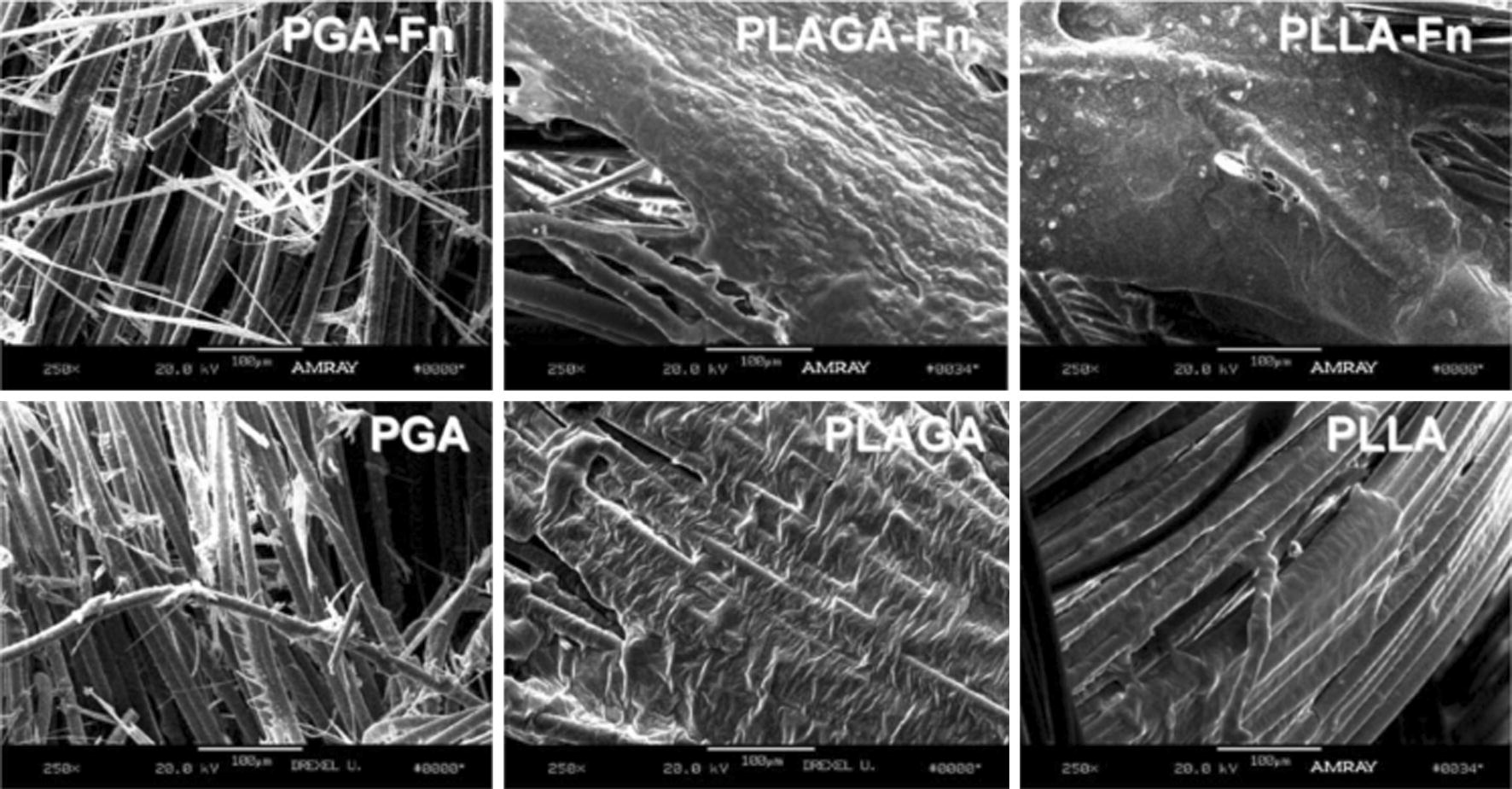
ACL fibroblast on braided scaffolds after 14 days of culture. Cells grown on braided scaffolds pre-coated with Fn elaborates a great amount of matrix compared to PLGA or PLLA scaffolds without Fn. Degradation of the PGA scaffold after 2 weeks of culture resulted in extensive cell loss and matrix depletion (Reprinted with permission [72])

In order to compare these three biomaterials, in addition to braided PLLA scaffolds, Lu et al. [72] also produced braided scaffolds made of PGA and PLGA to evaluate the effect of fiber composition on the mechanical properties and biodegradation. The scaffolds were coated with Fn before the culturing with primary rabbit ACL cells. Although PGA presented the highest tensile strength, the rapid degradation conducted to scaffold failure. Pre-coating the scaffold surfaces led to an increase in cell attachment efficiency and overall cell proliferation. Based on the overall cellular response, with highest rates of ACL fibroblast proliferation, and its superior mechanical and in vitro slow degradation properties, the PLLA braided scaffold coated with Fn was considered to be the most appropriate scaffold for ACL tissue engineering Fig. 7.

Wagner et al. [86] produced 3D porous polycaprolactone fumarate (PCLF) scaffolds to mimic the anterior cruciate ligament. Porous scaffold molds were designed using SolidWorks CAD software and 3D-printed. The scaffolds were produced by UV cross-linking of the PCLF solution and then seeded with human AMSCs in human platelet lysate. AMSCs proliferated, filling the pores and exhibited a collagen-rich extracellular matrix. At day 14, the cells remained viable and continued to increase in number, completely covering the surface and channels of the PCLF scaffold.

Table 4 presents a summary of the main studies that have used synthetic biodegradable polymers for ligament/tendon tissue engineering and the outcomes for the proposed scaffolds in terms of mechanical and in vitro/in vivo properties.

**Table 4:** Performance of synthetic biodegradable polymers in ligament/tendon TE

Material	Scaffold	Application	In vivo*/*in vitro	Mechanical response				Biological response	Refs.
				Young’s modulus (MPa)	Stiffness (N/mm)	Max. strength (MPa)	Max. load (N)		
PLGA	(i) Electrospun PLGA fibers onto knitted PLGA (ii) Knitted PLGA scaffold	Ligament/tendon	In vitro (porcine BMSCs)		(i) 0.85 ± 0.2 (ii) 0.64 ± 0.2 (at 14 days of culture)	ND		(i) Higher BMSCs attachment, proliferation, ECM deposition compared to (ii)	[71]
	Electrospun Aligned (i) Nanofiber diameter (320,680 nm) (ii) Microfiber diameter (1.8 µm)		In vitro (Human rotator cuff fibroblast)	(i) 421 ± 23 (ii) 510 ± 32 (for 1.8 µm)	ND	(i) ~ 13 (ii) ~ 14 (for 1.8 µm)	ND	(i) Higher cells proliferation (ii) Higher tendon-related ECM markers, after 14 days of culture	[87]
	Electrospun (i) Aligned nanofibers (ii) Random nanofibers	Tendon	In vitro (Human rotator cuff fibroblast)	(i) 341 ± 30 (ii) 107 ± 23	ND	(i) 12.0 ± 1.5 (ii) 3.7 ± 0.2	ND	Cellular adhesion, proliferation (i) Elongated morphology and orientation along the fibers (ii) Polygonal shape, random orientation	[70]
	(i) Rectangular braid (ii) Circular braid (scaffold with 3 regions)	Ligament	In vitro (fibroblasts: rabbit ACL; mouse BALB/C)	ND		(i) 217 ± 11 (ii) 212 ± 25	(i) 705 ± 36 (ii) 907 ± 132	Cellular adhesion and proliferation (i) ACL and BALB/C spread along the fibers (ii) Only ACL proliferate along the fibers	[6]
PLLA	Rectangular braid (scaffold with 3 regions)	Ligament/tendon	In vitro (rabbit ACL; MCL;AT; PT fibroblast)	ND				Cellular adhesion, proliferation, elongation along the fibers ACL with higher matrix production	[11, 74]
	Braided scaffolds of (i) 3, (ii) 4; (iii) 5 aligned electrospun nanofibers		In vitro (human MSCs)	(i) 55.0 ± 2.8 (ii) 47.8 ± 7.5 (iii) 47.6 ± 2.8	ND	(i) 7.62 ± 0.2 (ii) 6.57 ± 0.5 (iii) 6.67 ± 0.4	ND	(i) hMSCs adhesion and proliferation, orientation along the fibers; Expression of pluripotency genes (ii), (iii) ND	[88]
	(i) Braided scaffold (ii) Twisted fiber scaffold (iii) Braid-twist scaffold	Ligament	In vitro (rabbit PT fibroblasts)	(i) 810.2 ± 233.5 (ii) 888.2 ± 60.7; (iii) 428.2 ± 60.7	ND	(i) ~ 52.3 ± 7.7 (ii) 80.9 ± 6.9 (iii) 81.6 ± 1.6	ND	(i), (iii) Comparable cellular adhesion, proliferation. Production of ECM, after 7 days of culture (ii) ND	[75, 89]
	5 × 5 Square braid: (i) Scaffold before implantation (ii) Seeded (iii) Unseeded (i), (ii) 4 weeks post-surgery)		In vivo (after seeded with primary rabbit ACL cells) rabbit model	(i) 354.4 ± 68.5 (ii) 108.4 ± 27.7 (iii) 103.0 ± 53.9	ND		(i) 332.2 ± 19.6 (ii) 239.0 ± 43 (iii) 209.0 ± 73.5	(i) ND (ii) After 12 weeks, vascularization and greater tissue ingrowth and alignment of collagen fibers compared to (iii) A mild inflammatory response was observed in (i) and (ii)	[76]
	Electrospun (i) Aligned (ii) Random nanofibers	Tendon	In vitro/in vivo, (human tendon stem cells (hTSCs)) mouse model	(i) 22.76 ± 5.63 (ii) 0.63 ± 0.56	ND			hTSCs adhesion, proliferation In vitro/in vivo (i) cells with spindle-shaped and well orientated; teno-lineage differentiation (ii) Cells with round shapes; random distribution	[90]
(i) PLLA (ii) PLGA (iii) PGA (iv) Fn pre-coated: (i)–(iii)	Circular braid (multi-filament yarns-3 regions)	Ligament	In vitro (rabbit ACL fibroblasts)	ND		(i) 165 ± 33 (ii) 117 ± 12 (iii) 378 ± 18 (iv) ND	(i) 298 ± 59 (ii) 215 ± 23; (iii) 502 ± 24 (iv) ND	ACL adhesion, proliferation (i) Cells with spindle-like morphology (ii) Cells with a form of spiked rods (iii) Cells form large globular aggregates (iv) Higher ECM production; Fn improved cellular proliferation mainly on i)	[72]
PCLF	UV-crosslinked PCLF solution injected over 3D printed mold-square porous: (i) 500 × 500 µm (ii) 750 × 750 µm (mold designed by SolidWorks CAD software)	Ligament	In vitro (AMSCs)	ND				(i), (ii) AMSCs adhesion and proliferation, filling the pores, after 14 days of culture Expression of ligament-ECM in the presence of growth factors	[86]
PCL	Twisted electrospun fibers (i) Under static conditions (ii) Dynamic Loading	Tendon	In vitro (human MSCs)	(i) ~ 34 ± 6.8 (ii) ~ 27 ± 6.8 (acellular, after 21 days)	ND	(i) ~ 13 ± 0.7 (ii) ~ 12 ± 1.4 (acellular, after 21 days)	ND	Cellular adhesion/proliferation Elongated cells along the fiber direction (ii) Textured/round cells; Higher cell proliferation, comparing to (i) (i) Cells flatter and fused together (ii) Up-regulation of tendon genes, after 21 days	[91]

### Materials for ligament/tendon scaffolds

The difficulty of satisfying all the ideal scaffold requirements by using a single class of materials is a recurrent problem [39]. Advanced composite biomaterials have been fabricated to synergistically combine the beneficial properties of the constituents [39] and thus, achieving scaffolds that mimic complex structures of tendon/ligaments [92] and exhibit improved biological, biophysical and mechanical properties [2, 7, 12].

In the last years, the use of nanofillers (length < 100 nm) for the production of polymer nanocomposites has received great attention in academic research and industry. Even with low nanofiller content, nanocomposites exhibited unique properties compared to conventional composites [93, 94]. The significant higher surface-to-volume ratio of nanoparticles and their extremely higher characteristic ratio increase ductility with no decrease of strength and scratching resistance [95]. Besides, with the incorporation of nanoparticles in the polymer matrix, new properties may arise, which would not be possible when using macrosized particles [93]. Several nanocomposites with biodegradable polymer matrices have been developed specifically for various biomedical purposes such as drug delivery, tissue engineering, wound dressings, stem cell therapy and cancer therapy [94, 95]. The specific use of biodegradable polymer matrices for the production of the nanocomposites offers great advantages and include the ability to tailor mechanical properties and degradation kinetics to suit various applications [96]. Other advantages of using biodegradable matrices in TE approaches are their potential to fully restore the tendon or ligament tissues, with a simple surgical technique and minimal patient morbidity and risk of infection or disease transmission as well as rapid return to preinjury functions, by using biodegradable biomaterials scaffolds [1, 2, 12, 97].

#### Composites, blends and hybrid materials based on natural polymers

Scaffolds have been produced using collagen and sericin-extracted silk to improve scaffold properties for tendon/ligament applications and then seeded with cells [1, 98–102]. Chen et al. [54, 98] embedded MSCs derived from human embryonic stem cells within a knitted silk-Col sponge scaffold and achieved an enhancement of tendon tissue regeneration. They demonstrated through in vivo tests that dynamic mechanical stimulation is beneficial to tissue-engineered tendons, not only in terms of histology but also for the mechanical performance [98]. A similar silk-Col scaffold for MCL regeneration, seeded with MSCs had higher mechanical properties than a silk scaffold. The silk scaffold elicited a mild inflammatory reaction and degraded slowly after subcutaneous implantation in a mouse model [54]. Similarly, Shen et al. [99], Zheng et al. [100], Ran et al. [101] and Bi et al. [102] used scaffolds produced with Col micro-sponges in a knitted silk sponge matrix and all of them revealed efficient for tendon/ligament regeneration.

**Fig. 8:**
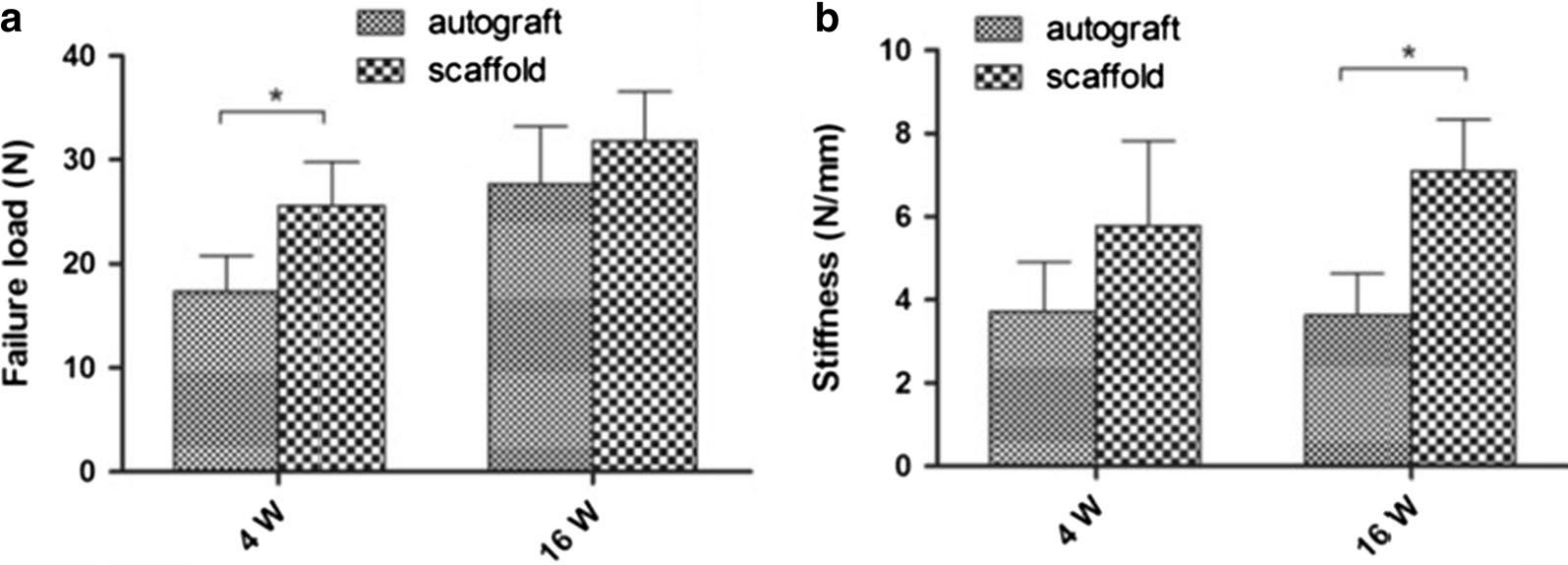
Statistical evaluation of differences in failure load (**a**) and stiffness (**b**) between the autograft group and scaffold group at 4 and 16 weeks postoperatively. *Significant difference between groups [102]

Bi et al. [102] evaluated the biomechanical performance of these silk-Col scaffolds and compared their performance with an autograft Fig. 8. Scaffolds were sterilized and implanted in vivo, in 20 rabbits, and autologous semitendinosus tendons were used to recover the ACL in the autograft control group. At 4 and 16 weeks after surgery, grafts were retrieved and analyzed. After 4 weeks of surgery, the failure load in the scaffold group was significantly higher than that in the autograft group (autograft, 17.33 ± 3.43 vs. scaffold, 25.63 ± 4.17 N; P < 0.05, n = 5). After 16 weeks, there was no significant difference in the failure load between the two groups (autograft, 27.64 ± 5.56 vs. scaffold, 31.85 ± 4.74 N, P > 0.05, n = 5; Fig. 8a). Regarding the stiffness, at 4 weeks postoperatively, there was no significant difference between the two groups (autograft, 3.72 ± 1.19 N/mm vs. scaffold, 5.78 ± 2.04 N/mm; P > 0.05, n = 5). However, at week 16, the stiffness in scaffold group was significantly greater than that of the autograft group (autograft, 3.63 ± 1.01 N/mm vs. scaffold, 7.09 ± 1.25 N/mm; P < 0.05, n = 5; Fig. 8b). Thus, the scaffold provided enough mechanical strength to resist the daily activities of the experimental rabbits [102].

ALG is an anionic polysaccharide. Its combination with CHI was reported by Majima et al. [61] and improves its biocompatibility and cell adhesive potential as well as decreases its degradation rate. This in vitro study using rabbit patellar tendon fibroblasts showed that ALG-0.1% CHI polyionic complex fibers had significantly higher cell attachment compared to ALG-only and polyglactin controls [61, 92].

In another study of Majima et al. [60], a biocompatible braided scaffold was produced from melt spun fibers of CHI and 0.1% HA. The scaffold presents adequate biodegradability and biocompatibility, with intense collagen type I production. The reduction in the strength of the composite fibers, due to water absorption, was measured after incubation for 0 h, 2 h, 14 days and 28 days in the standard culture medium. The tensile strength decreased after 2 h of incubation and then remained constant until 28 days Table 5. In vivo animal experiments with fibroblasts of Achilles tendon of a rabbit seeded on the CHI—0.1% HA hybrid-polymer fiber scaffold, showed that the mechanical properties of the scaffold had the possibility to stabilize the joint [60].

**Table 5:** Tensile strength of CHI-0.1% HA fiber after 0 h, 2 h, 14 days and 28 days in the standard medium (Dulbecco’s modified Eagle’s medium). Adapted from [60]

Incubation time	Tensile strength (MPa)
0 h	213.3 ± 10.0
2 h	60.0 ± 6.7
14 days	66.7 ± 6.8
28 days	65.1 ± 6.6

n = 45 in each sample (mean ± standard deviation)

A natural composite scaffold that combines silk, Col and HA was produced by Seo et al. [103] for ligament regeneration. In that study, a silk scaffold was knitted by hand and dry coated with collagen‐HA followed by freeze drying. The initial attachment and proliferation of human ACL cells on the composite silk scaffold was higher than the observed on the silk scaffold. The Col-HA substrate on the silk scaffold enhances new blood vessel and cell migration in vivo [103].

#### Composites, blends and hybrid materials based on natural and synthetic polymers

Natural materials have the advantage of being biocompatible, recognizable by cells, favoring the cell adhesion and proliferation. However, their quick degradability and low- mechanical properties may limit their application in tissue engineering, while synthetic polymers present low bioactivity and higher mechanical properties [37]. Thus, the combination both types of materials is expected to yield a synergetic effect between natural and synthetic polymers [37], and has been proposed as a good compromise between biological and mechanical performance for tendon and ligament regeneration [83, 104].

A hybrid scaffold comprised of degummed knitted silk microfibers coated with bioactive bFGF-releasing electrospun PLGA fibers was produced by Sahoo et al. [105] and its feasibility for use in ligament/tendon was evaluated in vitro. Rabbit BMSCs grew on PLGA fibers and silk microfibers and exhibited good viability. The release of bFGF stimulated cell proliferation and the gene expression of ligament/tendon-specific ECM proteins increased the collagen production and hence, the mechanical properties of the scaffold [105].

**Fig. 9:**
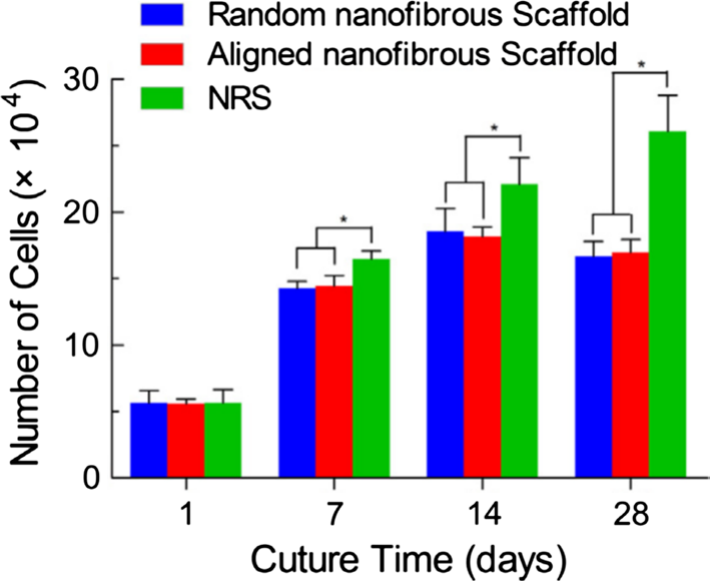
Commercially available cell counting kit-8 (CCK-8) result of MSCs cultured on the random nanofibrous scaffolds, aligned nanofibrous and NRSs for up to 28 days. The data are expressed as the mean ± SD. The samples marked with (*) has a significant difference between the two groups (P < 0.05) (Reprinted with permission from [106])

Three types of electrospun scaffolds of PLCL and silk fibroin, random nanofibrous scaffold, aligned nanofibrous scaffold and aligned nanoyarns (NRS), were studied by Yang et al. [106]. The Young’s modulus value of the NRS was lower than that of the aligned nanofibrous scaffold but was approximately two times higher than the one of the random nanofibrous scaffold. However, random and aligned nanofibrous scaffolds presented limitations in terms of cell infiltration due to the dense fiber packing. NRS configuration provided larger pores and enough space for cell infiltration which yielded improved cell proliferation for up to 28 days of culture as it can be observed in Fig. 9. NRS are used to achieve a balance between the porosity and mechanical properties of electrospun scaffolds [106].

Col has been widely combined with various polymers, often as a coating to stimulate tendon/ligament regeneration [80, 81, 92, 107]. Similarly to the previously reported work of Yang et al. [106], Xu et al. [107] studied three morphologies (random nanofiber, aligned nanofibers and aligned nanoyarn) of electrospun scaffolds composed by PLCL and in this case, collagen type I, for tendon tissue engineering. Nanoyarn scaffolds displayed desirable properties for tendon tissue engineering. Besides, tendon cells exhibited enhanced proliferation and expression of tendon-ECM genes on the nanoyarn scaffold, compared to random and aligned nanofiber scaffold [107].

**Fig. 10:**
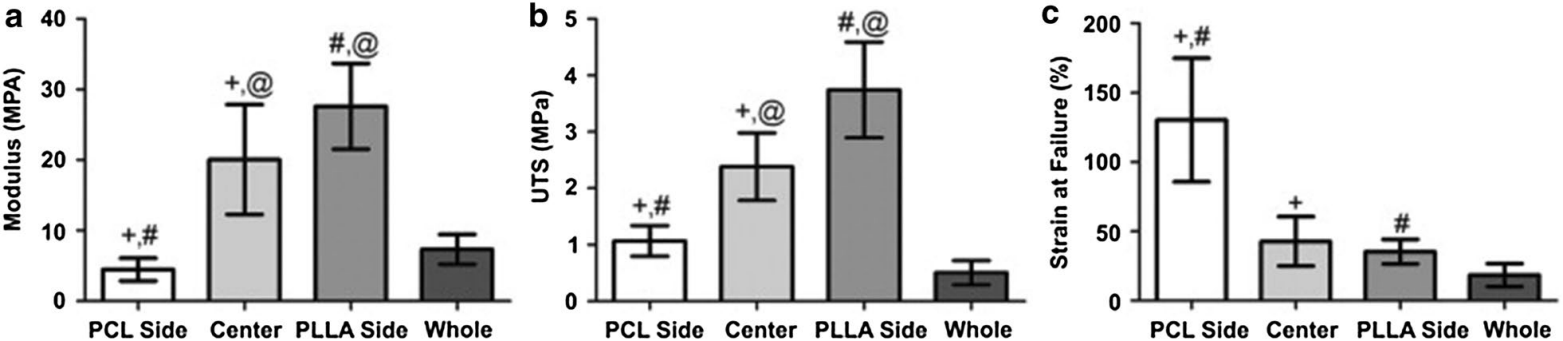
Average parameters obtained from tensile testing to failure of each region (*n* = 9) and the whole scaffold (*n* = 10). **a** Young’s modulus, **b** Ultimate tensile strength, **c** Strain at failure. +, #, @ indicate statistical significance with P < 0.05 (Reprinted with permission from [108])

Leong et al. [80] evaluated electrospun PCL grafts coated with Col, with and without the addition of bFGF and hFF, using an athymic rat model of ACL reconstruction. The histological and mechanical evaluation of PCL scaffolds demonstrated excellent healing and regenerative potential. After 16 weeks of implantation, Col + bFGF grafts presented the highest stiffness, achieving 58.8% of the stiffness and 40.7% of the peak load of healthy native ACL. The implantation of cells on the scaffolds does not appear to be beneficial for ligament regeneration while the implantation of bFGF had a beneficial effect on the graft cellularity and mechanical properties [80].

Similarly, Petrigliano et al. [84] used bFGF to treat PCL scaffolds (pre-coated with Col). Scaffolds were then seeded with BMSCs. Scaffolds treated with the growth factor and subjected to mechanical stimulation demonstrated cellular adherence and spreading at 21 days.

Electrospun bundles containing PLLA and collagen type I in different percentages, PLLA/Col-75/25 and PLLA/Col-50/50, were tested by Sensini et al. [104] to evaluate its potential for human Achille tendon regeneration. Human tenocytes were cultured over the same time range on the bundles and cell morphology was assessed. The mechanical properties (stiffness and strength) achieved are comparable to those of natural tendon. The PLLA/Col-75/25 blend was the most promising blend, with a Young modulus of 98.6 ± 12.4 MPa (as-spun), similar to that of native ligament and 205.1 ± 73.0 MPa, after 14 days in PBS. A good cell attachment and viability after 14 days of culture was observed. However, cells exhibited a better adhesion on PLLA/Coll-50/50 bundles and a more elongated morphology in comparison to PLLA/Coll-75/25 one [104].

A co-electrospun scaffold with 3 regions containing PCL-Col, a mixture of PLLA/Col and PCL/Col fibers and PLLA-Col was studied by Ladd et al. [108] for tendon–muscle junction tissue engineering. The scaffolds exhibited a randomly oriented nanofiber architecture in every region. The PLLA side had smaller fiber sizes on average, while the PCL side had larger fibers, and the center region, was a mixture of PLLA/Col and PCL/Col fibers with a fiber size in between. The scaffold was cytocompatible and accommodated cell attachment and myotube formation. Figure 10 shows the mechanical properties of this scaffold [108].

Sahoo et al. [81] reported the use of coating over PLLA and PLGA scaffolds, with PCL, PLGA nanofibers or collagen type I. They verified that collagen type I coating over both the PLGA or PLLA scaffolds offers a very favorable surface for MSCs attachment and proliferation. PLLA scaffolds exhibited reduced cell proliferation due to its hydrophobic character [81].

In order to study the ability to use nanomaterials to effectively reinforce collagen, Green et al. [109] produced gel-spun collagen type I and carbon nanofibers composite scaffolds, with 0.5% and 5% of filling load, for tendon tissue engineering. Fibers were subjected to fiber elongation and were crosslinked with glutaraldehyde. Wet-state tensile testing indicates that the structure and mechanical behavior are comparable to the native materials.

Other natural polymers such as CHI, ALG and HA have been combined with synthetic polymers [82]. For instance, Leung et al. [82] investigated aligned CHI–PCL nanofibers with TGF-b3 growth factor for tendon regeneration and they concluded that it led to a rapid and effective BMSCs differentiation into tenogenic progenitors [82]. Domingues et al. [83] reported the use of cellulose nanocrystals as reinforcing agents in aligned electrospun scaffolds containing PCL and CHI. The nanocomposite fibrous scaffolds fulfill the mechanical requirements for tendon TE applications and the aligned morphology promoted a remarkable uniaxial cell orientation and induced elongated cell morphology [83]. A PLCL (lactic acid/ε-caprolactone proportion of 85/15) multilayered braided scaffold was produced by Liu et al. [69] A layer-by-layer coating was introduced by immersing the scaffolds into poly-l-lysine solution (polycation) and subsequently into HA solution (polyanion) to promote MSCs growth, differentiation, and migration. The braided PLCL scaffold with one-layer of poly-l-lysine and HA modification shows biocompatibility and satisfying mechanical properties that may constitute a promising scaffold for ligament tissue engineering [69].

#### Composites, blends and hybrid materials based on synthetic polymers

**Fig. 11:**
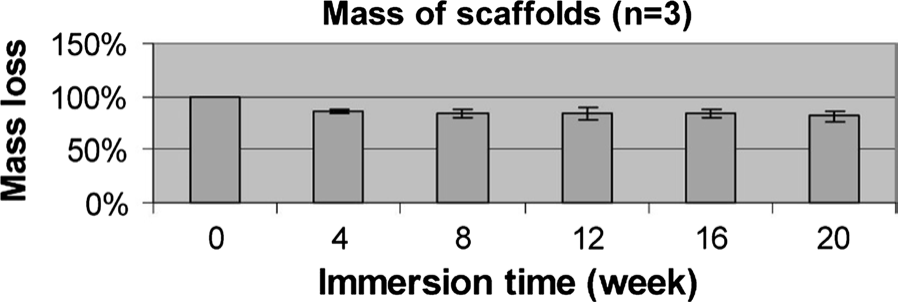
Mass loss of knitted structure during 20 weeks [35]

The combination of different synthetic polymers has also been a strategic design for achieving hybrid scaffolds for ligament/tendon regeneration. For instance, although PLGA exhibits good cell affinity, it also presents a rapid degradation which limits its application in tissue engineering. For that reason, PLGA may be combined with another material with slower degradation rate, such as PLLA to ensure the scaffolds’ integrity and adequate mechanical properties for a longer time. A PLLA-PLGA knitted scaffold was studied for ligament tissue engineering by Ge et al. [35]. To understand the degradability of the biomaterial, in vitro degradation tests were performed, by immersing the knitted scaffolds in cell-culture medium for 20 weeks. As can be seen in Fig. 11, there was obvious mass loss at initial 4 week. This is possibility attributed to relatively quick degradation of PLGA, which may be important to promote potential tissue in-growth, at the initial stage of implantation. Comparing to PLLA yarns, PLGA yarns degraded more quickly and were not visible at 8 weeks. PLLA yarns kept their integrity for at least 20 weeks [35]. They found that this scaffold can fulfill most of the requirements in terms of porosity, degradation rate and mechanical properties [35]. When seeded onto these scaffolds, MSCs proliferated and increased the synthesis of collagen type I and type III [110].

**Fig. 12:**
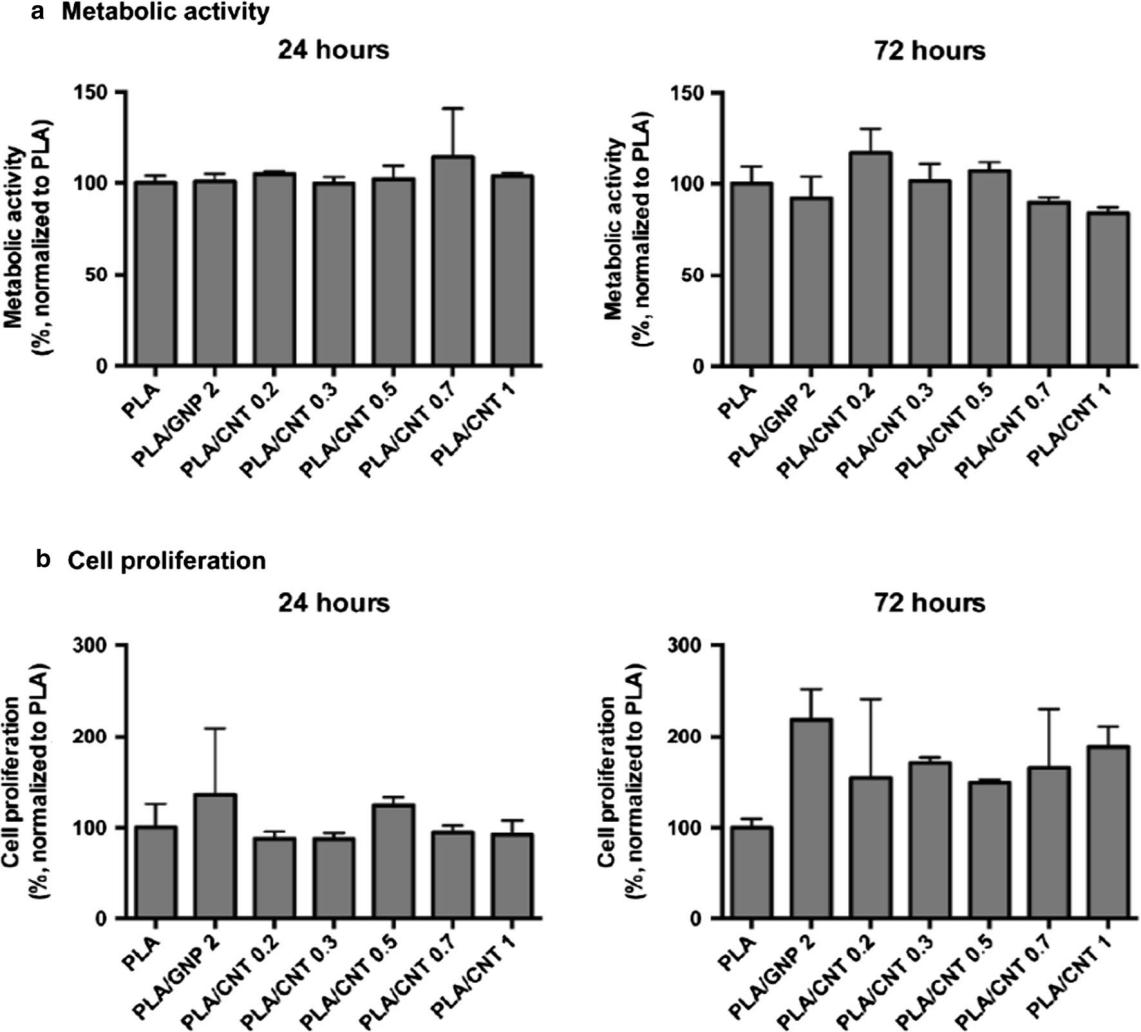
Viability (**a**) and proliferation (**b**) of fibroblasts seeded in different composites after 24 and 72 h in culture. Results are normalized with respect to the values for cells cultured in PLA control (Reprinted with permission from [111])

Pinto et al. [111] reported the production of nanocomposite thin films containing PLA/COOH functionalized carbon nanotubes (CNT-COOH) and PLA/graphene nanoplatelets (GNP). In vitro tests were performed by seeding human dermal fibroblasts (HDF) onto PLA, PLA/GNP and PLA/CNT-COOH films and all formulations exhibited no cytotoxic responses and supported cell proliferation up to 3 days in culture. After 72 h of in vitro culture, HDF exhibited higher proliferation on the nanocomposite materials with PLA/CNT 0.3% and PLA/CNT 0.5%, when compared to PLA. Besides, increasing percentages of CNT-COOH within PLA matrix did not affect cultured fibroblasts Fig. 12. In vivo tests performed by subcutaneous implantation of nanocomposites in mice showed no severe inflammatory response, as observed 1 and 2 weeks after implantation, which supports that the use of carbon-based nanofillers in PLA-based structures has potential for ACL reinforcement [111].

In a previous study Pinto et al. [112] reported that the carbon nanostructures improved the mechanical properties of the PLA composites, approaching the range of natural tendons and ligaments: tensile strength in the range of 5–100 MPa and Young’s modulus from 20 MPa to 1200 MPa [113]. The composite with 0.7 wt% CNT-COOH presented enhanced tensile strength relative to PLA (from 59.90 ± 4.93 MPa to 72.22 ± 1.52 MPa), as well as elongation at break (from 1.86 ± 0.06% to 2.25 ± 0.40%) [112]. Besides, the composites with 0.7 wt% CNT-COOH and 2 wt% GNP showed a considerable increase (> 20%) in the Young’s modulus relative to PLA, from 3.99 ± 0.42 GPa to 4.86 ± 0.47 GPa and 4.92 ± 0.15 GPa, for PLA-CNT-COOH and PLA-GNP, respectively. The composite scaffolds were cytocompatible, supporting fibroblasts metabolic activity and proliferation up to 72 h [112].

Liu et al. [114] produced a 3D biodegradable PLA screw-like scaffold coated with hydroxyapatite for ACL regeneration. The scaffold presented adequate size porosity and the pores were interconnected in regular patterns with orthogonal structure. MSCs were seeded on PLA scaffold, PLA-hydroxyapatite scaffold, and suspended in Pluronic F-127 hydrogel on PLA-hydroxyapatite scaffold. The last group showed the highest in vitro cell proliferation and osteogenesis. For the histological examination, PLA, PLA-hydroxyapatite, and PLA-hydroxyapatite loaded MSCs screw-like scaffolds were implanted into the femoral tunnel of rabbits. The histological results revealed that PLA-hydroxyapatite scaffolds with MSCs seeded presented increased new bone formation at the interface between the bone tunnel and graft after 12 weeks. Hydroxyapatite surface modification not only enhanced new bone ingrowth but also the proliferation and migration of MSCs and osteoblasts with excellent vascularization [114].

Sahoo et al. [81] reported that coating PLLA or PLGA scaffolds with collagen type I also offers a very favorable surface for MSCs attachment and proliferation. However, they verified that compared to Col, a PCL coating on PLLA or PLGA scaffolds resulted in a reduced cell attachment and higher mechanical strength [81].

**Fig. 13:**
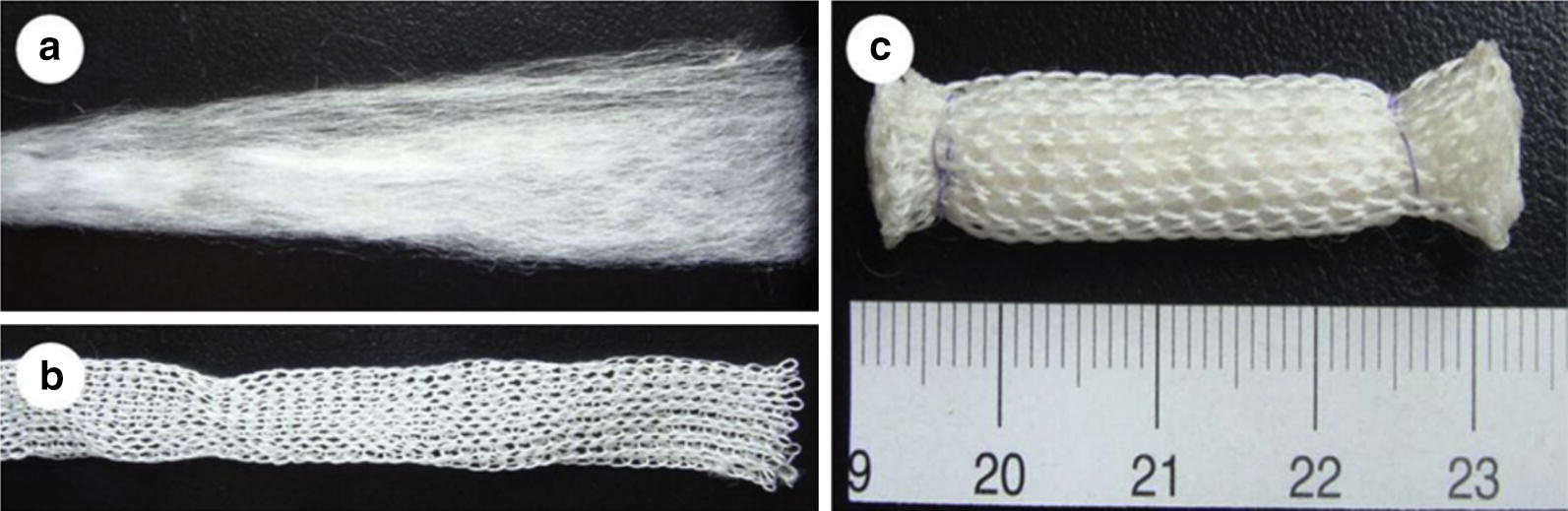
Preparation of a composite tendon scaffold. The scaffold was composed of an inner part of PGA unwoven fibers (**a**) and an outer part of a net knitted with PGA/PLA fibers in a ratio of 4:2 (**b**). The outcome of assembled two parts (**c**) (Reprinted with permission from [115])

A composite tendon scaffold composed of an inner part of PGA unwoven fibers and an outer part of knitted PGA/PLA fibers, to provide mechanical strength, was produced by Deng et al. [115], Fig. 13.

**Fig. 14:**
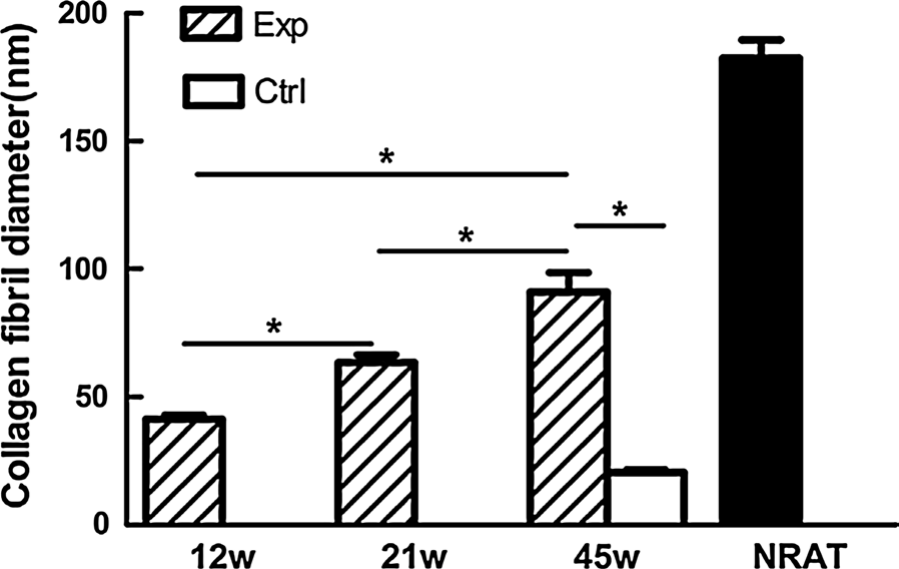
Quantification of collagen fibril diameter of in vivo engineered tendons with native tendon as a control. Collagen fibril diameter of in vivo engineered tendons increased with time. There was significant difference between 12 and 21 weeks, between 21 and 45 weeks and between 12 and 45 weeks of the AMSCs seeded group (*P < 0.001). There was significant difference between two groups at 45 weeks post implantation (*P < 0.001). *Exp* experimental group, *Ctrl* control group, *w* week, *NRAT* normal rabbit tendon (Reprinted with permission from [115])

AMSCs were seeded onto these scaffolds [115]. Cytocompatibility between cells and PGA fibers was found since short-term in vitro culture enabled AMSCs proliferation and the production of extracellular matrix on the PGA fibers. The scaffolds exhibited a tensile strength around 50 MPa [115]. The in vitro cultured scaffolds were then subjected to an in vivo transplantation on rabbits. Cell-seeded scaffold was integrated within the native tissue and with the increase of implantation time, cells gradually form neo-tendon. The diameter of collagen fibrils significantly increased which is related to the role of seeded AMSCs in the formation of engineered tendon in vivo Fig. 14. After 45 weeks of implantation, there was no obvious remaining scaffold-base material and the formed tendon exhibited a cord-like shape with a smooth surface, comparable to the normal tendon [115].

A summary of the studies that have used composites, blends and hybrid materials based on natural or synthetic polymers for tendon/ligament TE are presented in Tables 6 and 7, respectively. A review about composites, blends and hybrid materials based on the combination of natural and synthetic polymers for tendon/ligament TE is presented in Table 8. These tables include, for each combination of materials, the proposed scaffold and the reported mechanical and in vitro/in vivo properties.

**Table 6:** Performance of composites, blends and hybrid materials based on natural polymers for ligament/tendon TE

Material	Scaffold	Application	In vivo*/*in vitro	Mechanical response				Biological response	Refs.
				Young’s modulus(MPa)	Stiffness (N/mm)	Max. strength (MPa)	Max. load (N)		
Silk-Col	Col -sponge incorporated into a freeze-dried knitted silk mesh (i) addition of: rhSDF-1 alpha (ii) no rhSDF-1 alpha^a^	Ligament	In vivo, rabbit model	ND	7.02 ± 1.25 (at 16 weeks after surgery)	ND		Fibroblasts distribution throughout the scaffold, at 4 weeks post-implantation At 16 weeks postoperatively, host cells had invaded the core of the scaffold	[102]
				49.49 ± 10.14 (at 16 weeks post surgery)		ND	~ 25 ± 12	At 2 weeks post-reconstruction, spindle tendon-like cells and vascularization At 12 weeks, ligament-ECM deposition	[101]
		Ligament/tendon	In vitro (BMSCs); In vivo, rabbit model	ND	24.3 ± 2.9	ND	47.0 ± 7.4	At 15–360 days after implantation, tissue ingrowth by fibroblasts between the fibers At 4 weeks, denser ECM, larger number of cells, spindle-shaped morphology Rarely vascularity; no evident inflammation	[54]
			In vitro (human embryonic-stem cells MSCs) In vivo, mouse model (i) Dynamic mechanical stress (ii) No mechanical stress	24.44 ± 10.03 (unseeded)	ND			In vitro: MSCs adhesion, proliferation (i) After 14 days, cells elongation, aligned along the direction of mechanical stress In vivo: (i) Aligned cells and larger collagen fibers comparing to (ii) After 4 weeks post-surgery, tendon-related ECM, indicating tenocyte-lineage	[98]
		Tendon	In vitro (BMSCs, HDF), Achilles tendon fibroblasts (ATFs) *In vivo*, rat model^a^	(i) 45.3 ± 10.4 (ii) 32.6 ± 3.9^a^	ND		(i) 68.5 ± 18.0 (ii) 65.7 ± 10.3^a^	(i), (ii) At 4 days post-surgery neo-tendons appeared with cord-like shape^a^ (i) Migratory BMSCs/HDF; vascularization; at 1 week post-surgery more fibroblasts and ECM; At 4 weeks, organized bundles of collagen fibers	[99]
CHI-HA	Braid with wetspun fibers	Ligament/Tendon	In vitro (fibroblast rabbit PT) (i) static (ii) stretch (iii) rotation (iv) stretch+ rotation	ND				Cell adhesion and proliferation (iv) Higher cell proliferation after 21 days of culture and ECM production after 14 days of culture, comparing to i-(iii) (iii) Higher cell proliferation than (i) and (ii)	[117]
Silk coated with Col-HA	(i) Knitted silk (ii) Freeze-dried silk coated with Col-HA	Ligament	In vitro (Human ACL fibroblasts); In vivo, dog model	ND				In vitro: (ii) Higher cell attachment, proliferation and ECM synthesis than (i) In vivo: (i), (ii) Synovitis; (ii) induced angiogenesis, new collagen formation and higher vascularity than (i)	[103]

^a^rhSDF-1 alpha(exogenous recombinant human SDF): cytokine that regulates stem cell homing

**Table 7:** Performance of composites, blends and hybrid materials based on synthetic polymers for ligament/tendon TE

Material	Scaffold	Application	In vivo*/*in vitro	Mechanical performance			Biological performance	Refs.
				Young’s modulus(MPa)	Max. strength(MPa)	Max. load (N)		
PGA-PLA	Two ends cord: inner part: PGA unwoven fibers outer part: knitted PGA and PLA fibers	Tendon	In vitro (AMSCs); in vivo, rabbit model	ND	53.71 ± 22.32	ND	AMSCs adhesion and proliferation, ECM synthesis At 12 weeks post-repair, middle part with organized Col pattern and host inflammatory cells At 45 weeks, mature elongated tendons, aligned Col fibers; Scaffold completely degraded	[115]
PLA-GNP; PLA-(CNT-COOH)	Melt-blended films of: (i) PLA/CNT-COOH (ii) PLA/GNP	Ligament/tendon	In vitro (HDF), in vivo, mice model	(i) 4860 ± 470 (ii) 4920 ± 150	(i) 72.22 ± 1.52 (ii) 58.56 ± 3.99	ND	In vitro: Cell adhesion, proliferation; (i) Higher proliferation compared to (ii) In vivo: localized inflammatory response Livers with no toxicity for (i), (ii)	[111, 112]
PLLA-PLGA	(i) PLLA yarns (ii) PLGA yarns (iii) Two ends Knitted (PLLA-PLGA)	Ligament	ND	(iii) ~ 287	(iii) ~ 60	(iii) 72	ND	[35]
	Two ends Knitted (PLLA-PLGA): (i) no cells, no fascia lata wrap (ii) MSCs seeded (iii) MSCs seeded + fascia wrap (iv) fascia lata wrap	Ligament	In vivo, rabbit model	ND	ND	(i) 14.0 ± 7.8 (ii) 14.9 ± 6.6 (iii) 20.9 ± 4.5 (iv) 15.8 ± 6.8 (at 20 weeks post-surgery)	(i)–(iv) Cellular spread and elongation; few macrophages; ECM synthesis (i), (ii) Non parallel fibroblasts (iii), (iv) Higher Col type I and type III	[110]
PLLA-PEGDA^a^	(i) Braid-twist fibrous PLLA and crosslinked PEGDA hydrogel (ii) Braid twist (no hydrogel)	Ligament	In vitro (primary rabbit PT fibroblasts)	(i) 437 ± 38 (ii) ND	(i) 36 ± 3 (ii) ND	ND	(i) Higher cell proliferation than (ii), at day 14 of culture (i), (ii) Comparable cell proliferation, at day 21 and 28 of culture	[118]
PLA-Hydroxyapatite	3D printed PLA scaffold: (i) No coating (ii) Coated with hydroxyapatite (iii) Coated with hydroxyapatite + MSCs suspended in hydrogel	Ligament	In vitro (rabbit MSCs); in vivo, rabbit model	ND			In vitro: (iii) Higher cell proliferation and osteogenic markers, compared to (i), (ii) In vivo: (iii) After 4 weeks post-surgery, more chondrocytes and cartilage matrix in the interface with the bone; higher Col fibers, after 12 weeks	[114]

^a^Polyethylene glycol diacrylate (PEGDA)

**Table 8:** Performance of composites, blends and hybrid materials based on natural and synthetic polymers for ligament/tendon TE

Material	Scaffold		Application	In vivo/in vitro	Mechanical performance				Biological Performance	Refs.
					Young’s modulus (MPa)	Stiffness (N/mm)	Max. strength (MPa)	Max. load (N)		
CHI-PCL-cellulose	Electrospun CHI-PCL nanofibers + cellulose nanocrystals: i) Aligned nanofibers (ii) Random nanofibers		Tendon	In vitro (human tenocytes)	(i) 540.5 ± 83.7 (ii) ND	ND	(i) 39.3 ± 1.9 (ii) ND	ND	Cellular adhesion/spread (i) Cells elongated/aligned along the nanofibers (ii) Cells with random shape and orientation Synthesis of tendon-specific markers	[83]
Silk-PLCL	Electrospun (i) Aligned nanoyarn-reinforced random fibers (NRS) (ii) Random nanofibers (iii) Aligned nanofibers			In vitro (primary rat BMSCs)	(i) 288.95 ± 13.26 (ii) 186.65 ± 8.87 (iii) 433.56 ± 48.06	ND	(i) 24.25 ± 0.76 (ii) 9.70 ± 0.51 (iii) 39.10 ± 2.89	ND	BMSCs adhesion/spread (i) Higher cell spread than (ii), (iii); cells elongation/random distribution (ii) Cells with random distribution, pyramidal shape (iii) Cellular elongation	[106]
Col-PLCL	Electrospun (i) Nanoyarn (ii) Random nanofibers (iii) Aligned nanofibers			In vitro (primary rabbit tendon cells)	(i) ~ 2.1 (ii) ~ 3.9 iii) ~ 4.5	ND	(i) ~ 3.3 (ii) ~ 5.6 (iii) ~ 6.2	ND	Cell adhesion, spread (i) Higher cell growth (i), (ii) Elongation along the nanofibers/nanoyarn (iii) Cells with random spread (i) Higher tendon-ECM genes, compared to (ii), (iii), after 14 days	[107]
Col-PD^a^	Electrospun (i) Col nanofibers (ii) Col microfibers coated with PD		Tendon	In vivo, rabbit model	(i) 0.549 (ii) 0.754 (60 days post surgery)	(i) 20.37 ii) 29.87 (60 days post surgery)	i) 10.44 (ii) 11.37 (60 days post surgery)	(i) 52.72 (ii) 74.02 (60 days post surgery)	(i), (ii) Some inflammatory response, after surgery (ii) Cells with better alignment and higher mature tenoblasts and macrophages, compared to (i), 60 days post-surgery; Scaffold partially degraded	[119]
Col-Carbon nanofibers	Elongated gel-spun fibers: (i) Col/ 0.5 carbon nanochips crosslinked with glutaraldehyde (ii) Col/ 0.5 carbon nanochips (iii) Col/ 0.5SWNTs^b^, crosslinked with glutaraldehyde (iv) Col/ 0.5SWNTs			ND	(i) 590 ± 50 (ii) 46 ± 4 (iii) 840 ± 40 (iv) 92 ± 35 (wet-state)	ND	(i) 75 ± 15 (ii) 5 ± 2 (iii) 70 ± 8 (iv) 9 ± 1 (wet-state)	ND	ND	[109]
(PLLA-Col)-(PCL-Col)	Co-electrospun onto opposite ends: PLLA-Col PCL-Col (3 regions)			In vitro (myoblastsand fibroblasts)	7.34 ± 2.13	ND	0.51 ± 0.21	ND	Myoblasts and fibroblasts adhesion/proliferation onto the 3 regions Myoblasts differentiation into myotubes	[108]
Silk coated with PLGA	Knitted silk microfibers coated with	Random electrospun PLGA nanofibers	Ligament/tendon	In vitro (rabbit BMSCs)	ND	4.8 ± 0.52	ND		Cellular adhesion, proliferation Production of ECM between the nanofibers	[120]
		(i) bFGF-releasing electrospun PLGA nanofibers (ii) Electrospun PLGA nanofibers			ND	(i) 4.3 ± 0.3 (ii) ND	ND		Cell adhesion/spread (i) Higher cell proliferation, viability; higher Col production, ligament/tendon-specific ECM, from day 7 to 14, comparing to (ii)	[105]
Silk coated with PCL or P3HB^c^	Twisted nanofiber-coated silk yarn Nanofiber coating: (i) PCL (ii) P3HB (iii) Electrospun PLGA nanofibers			In vitro (L929 murine fibroblasts)	ND			(i) 110.5 ± 6.6 (ii) 97.6 ± 11.4 (iii) 92.6 ± 8.2	Cell adhesion/spread Cell viability decreased from the 1st to 3rd day of culture; (i), (ii) Higher cell viability than (iii), after 3 days of culture	[121]
PCL coated with Col	Electrospun PCL scaffold coated with: (i) Col (ii) Col + bFGF (iii) Col + hFF (iv) Col + bFGF + hFF		Ligament	In vivo, rat model	ND	(i) 12.4 ± 3.8 (ii) 23.3 ± 8.1 iii) 4.4 ± 1.2 iv) 10.1 ± 2.1 (16 weeks post-surgery)	ND	(i) 16.0 ± 3.4 (ii) 23.1 ± 6.1 (iii) 17 ± 6.9 (iv) 15.1 ± 4.9 (16 weeks post-surgery)	Cell proliferation and alignment along the fibers; Col deposition (ii) Higher cell proliferation than (i), at 16 weeks post-surgery (iii), (iv) No beneficial effect of hFF for regeneration	[80]
PLCL (85/15)-poly-l-lysine-HA	Multilayer braid: (i) PLCL (ii) PLCL+Poly-l-lysine iii) PLCL + poly-l-lysine + HA			In vitro (human BMSCs and WJ-MSCs^d^)	(i) 1616 ± 643 (ii) 1608 ± 156 (iii) 1758 ± 470	ND			Cell adhesion, proliferation, elongation and alignment; ECM synthesis on day 14; Metabolic activities decreased from (i) to (ii) and (iii) (iii) Higher MSCs migration	[69]
PLLA-gelatin-Col	Gelatin-bFGF hydrogel sandwiched by Fn-coated PLLA braided scaffold, and wrapped with a Col membrane, reinforced with PLLA microspheres			In vivo, rabbit model	ND	~ 30	ND		At 8 weeks post-surgery, great cell spread/migration; vascularization induced by bFGF; great Col and ECM synthesis	[38]

^a^Polydioxanone(PD); ^b^Single walled nanotubes (SWNTs); ^c^poly(3-hydroxybutyrate) (P3HB); ^d^Wharton’s jelly mesenchymal stem cells (WJ‐MSCs)

## Processing techniques of ligament/tendon scaffolds

The architecture of the scaffold is an important design concern since it can modulate the mechanical and biological response and hence, determine the long-term clinical success of the scaffold [33]. Literature has reported several methods to produce tendon/ligament scaffolds including gas foaming, phase separation, emulsion freeze-drying and porogen leaching [66]. However, their ability to precisely control the pore size and interconnectivity as well as scaffolds’ structure and mechanical properties is often limited [116].

**Fig. 15:**
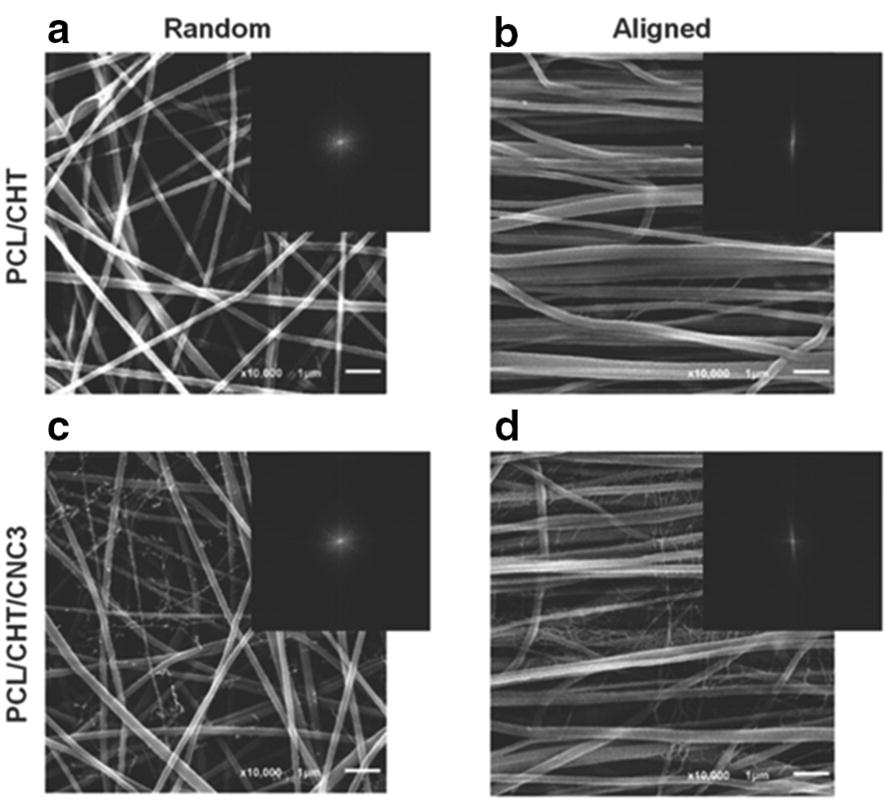
SEM images of random nanofiber meshes and aligned nanofiber bundles of (**a**, **b**) PCL/CHI and (**c**, **d**) PCL/CHI/ cellulose nanocrystals (3wt.%) with the respective 2D-fast Fourier transform frequency plots. Scale bar 1 μm (Reprinted with permission from [83])

Since both tendons and ligaments are fibrous tissues, the production of fiber-based scaffolds has been the preferred option for tendon/ligament TE and has proven to promote cellular proliferation and collagenous matrix deposition [9, 33]. The main factor is the way that fibers are organized. Parallel align of fibers/yarns is the simplest way to organize fibers [9] and has been widely reported for tendon TE approaches [8, 14, 83, 106, 107]. These fibers are commonly achieved through electrospinning [83, 106, 107] or electrochemical alignment [62, 122]. Figure 15 illustrates scanning electron micrographs of (A) aligned and (B) random nanofiber scaffolds proposed by Domingues et al. [83] for tendon regeneration. However, the lack of interaction between the fibers usually restrict its application [8]. Attending to the complexity of the ligament/tendon, the most common approach adopted by researchers relies on complex structures produced by textile techniques [116], in which fibers are engineered into braided, knitted, twisted or woven structures to obtain hierarchical scaffolds [8, 9, 14].

**Fig. 16:**
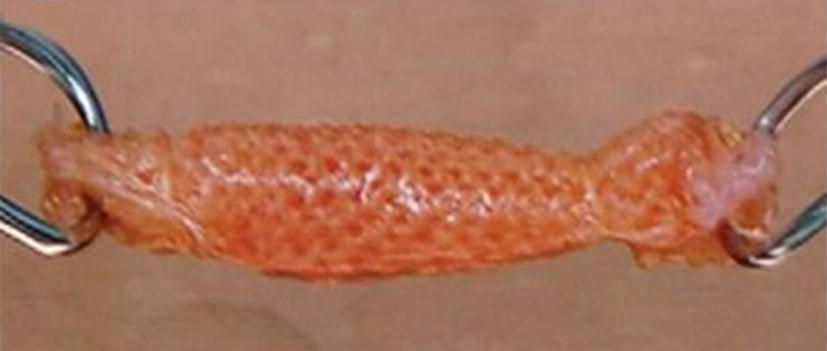
BMSCs-seeded (7 days of culture) scaffold produced by electrospinning bFGF-PLGA fibers onto the surfaces of knitted microfibrous silk scaffolds [105]

Electrospinning allows the production of long continuous fibers with controlled diameter ranging from nanometers to microns, mimicking the nanoscale structure of tendon and ligament ECM [9]. It allows the production of fibers from several natural and synthetic polymers including Col, CHI, HA, silk fibroin [9] or PCL [91], PLGA [71], PLA [90], as well as combinations of natural and synthetic fibers [80, 83]. However, the weak mechanical properties of the electrospun scaffolds produced for tendon/ligament TE limit the successful translation to the clinic [34]. Additionally, electrospinning typically produces 2D fiber mats, limiting the production of 3D hierarchical structures. For that reason, electrospun nanofibers have been twisted or rolled using standard textile techniques such as e.g. weaving or braiding, to produce 3D hierarchical structures with proper mechanical properties [34]. Yarns made of aligned fibers can be formed by electrospinning and then intertwined to form braided [88] or knitted scaffolds as can be observed in Fig. 16 [105].

Textile technologies allow the production of complex 3D constructs from monofilaments and multifilament threads, for various TE applications, being extensively applied in tendon/ligament regeneration [116]. These scaffolds are produced by several textile methods such as braiding, twisting, wire-rope, weaving and knitting [52] that enable tailoring the scaffolds’ architecture by controlling the fiber size/orientation, pore size and interconnectivity, surface topography, mechanical properties and the cellular distribution that scaffold provides [116].

**Fig. 17:**
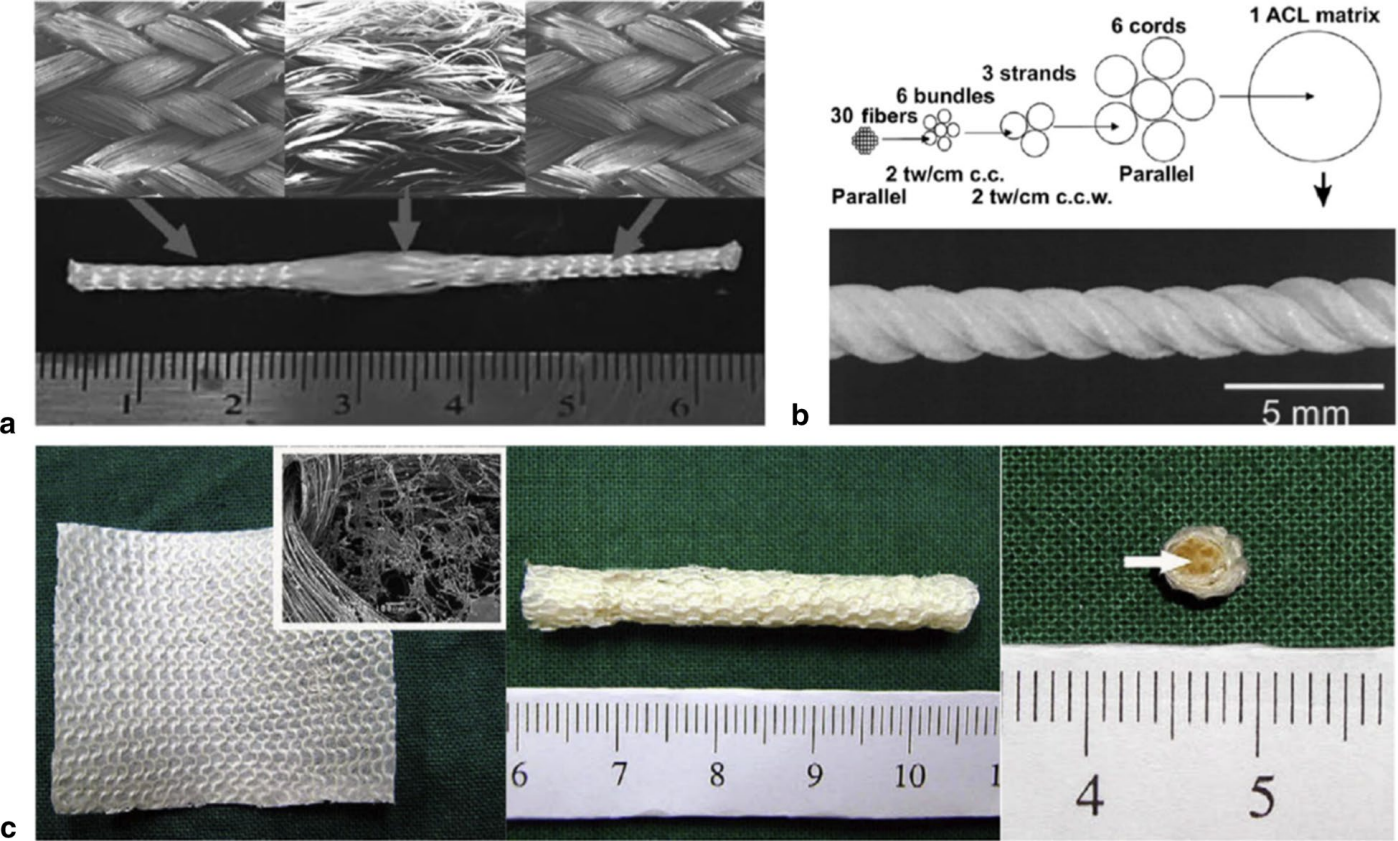
Scaffolds for ligament tissue engineering. **a** Braided scaffold with a fibrous intra-articular zone terminated at each end by a less porous bony attachment zones in a single braid; **b** twisted fibrous scaffold; **c** silk scaffold produced by rolling up the porous knitted silk mesh around a silk cord (Reprinted with permission from [34])

Twisted scaffolds are formed with multilevel yarns that combine multiple ends at a single point and twisting the structure together [52]. Twisted structures ensure interaction between fibers, unlike parallel aligned fibers, and are morphologically closer to native ligament, as depicted in Fig. 17b [8].

Knitting allows the production of complex structures from a yarn that is interlaced in a previous loop to form interconnected loops. Knitted scaffolds present different mechanical and physical properties depending on the type of stitches and the yarn material. While the production of knitted structures with adjustable properties in different directions is difficult, it is possible to produce 3D structures with precise microstructure control by combining knitting machines with computer-aided design (CAD) systems [116]. Knitted scaffolds for tendon/ligament tissue engineering [9, 103, 110] have demonstrated good mechanical properties and adequate porosity for tissue ingrowth Fig. 17c [71].

**Fig. 18:**
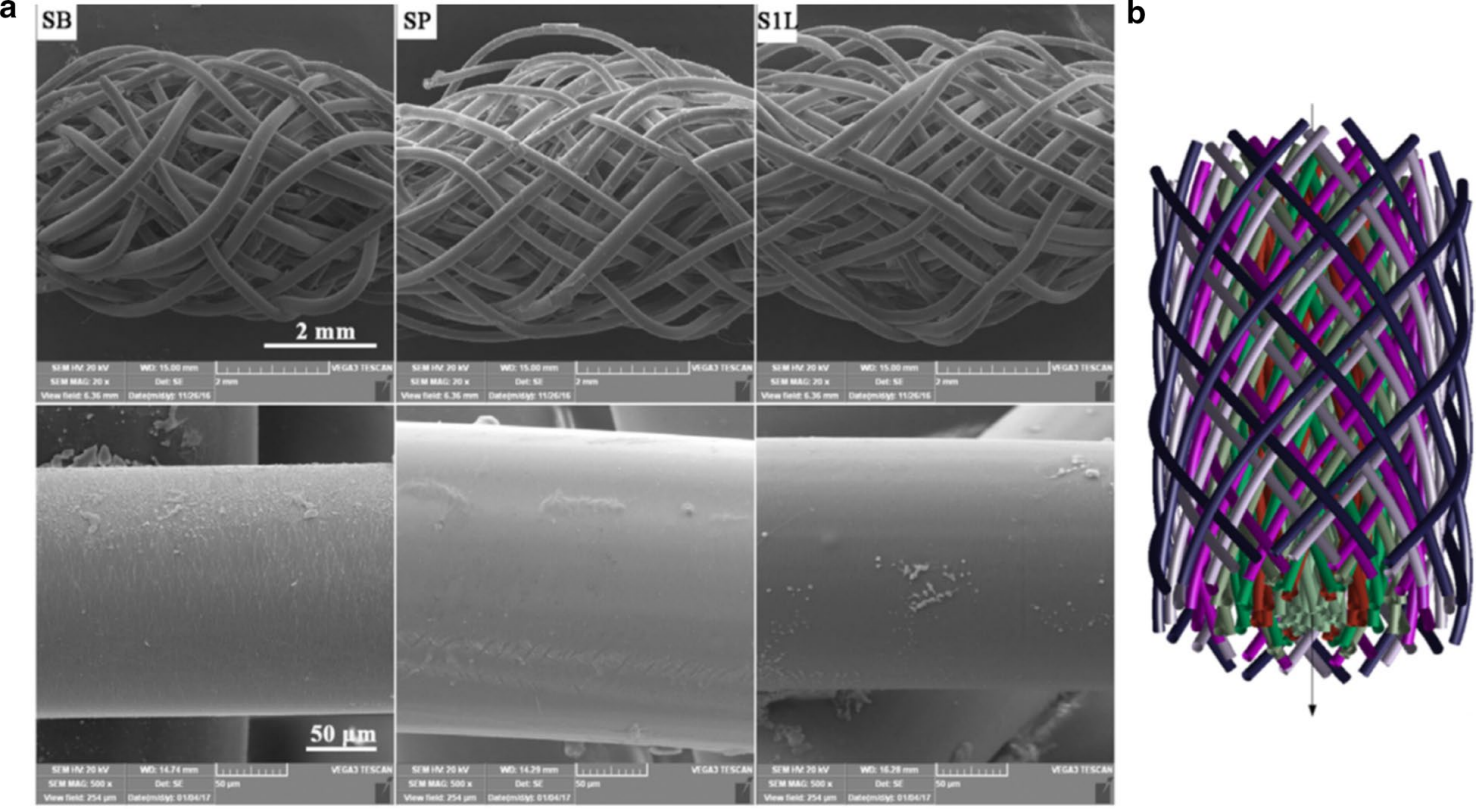
**a** Morphology of PLCL scaffold and PLCL scaffold modified with poly-l-lysine and HA by scanning electron microscopy (SB: scaffold blank; SP: PLCL-poly-l-lysine; S1L: PLCL-poly-l-lysine/HA-PLCL-poly-l-lysine). **b** Global structure of the multi-layer braided scaffold. The six different constitutive layers, made of 16 fibers/layer, are represented with different colors (Reprinted with permission from [69])

Braiding technique comprises three or more yarns intertwined in overlapping patterns [116]. In general, braided scaffolds are dimensionally very stable, having good flexibility, high strength and fatigue resistance [8]. These enhanced mechanical properties promoted their extensive application in tendon and ligament scaffolds with biomimetic characteristics [116]. The morphology of the braided scaffolds made of PLCL and modified PLCL developed by Liu et al. [69] are illustrated in Fig. 18a as well as the global structure of the multilayer braided scaffolds (B). The mechanical and biological properties of these scaffolds were reported above.

Braided structures present low porosity which restricts the tissue ingrowth [8], as compared to the highly porous knitted structures that favor tissue ingrowth and the deposition of collagenous connective tissue, which is crucial for tendon/ligament reconstruction [8, 71]. The pore size of braided structures may be controlled by varying the yarn size and braiding angle, which may also develop anisotropic mechanical properties with adjustable gradient along any desired direction [116]. Laurencin et al. [11] proposed a braided scaffold for ACL regeneration that comprised three regions: femoral tunnel attachment site (bony attachment end), ligament region (intra-articular zone), and tibial tunnel attachment site (bony attachment end) as illustrated in Fig. 17a. The attachment sites exhibit a high-angle fiber orientation and smaller pore size to improve the quality of anchorage in bone tunnels and provide resistance to wear within it. The intra-articular zone (central region with larger pore size) has a lower-angle fiber orientation. A minimum pore diameter of 150 µm is suggested for bone and 200–250 µm for soft tissue ingrowth [11].

Researchers have reported the production of yarns made of twisted fibers combined by the braiding process [75] in order to withstand it, since the degree of twisting as well as the direction affect the yarn strength, abrasion resistance, and flexibility [33]. Table 9 presents the braiding and twisting angles associated to each braided scaffold, braided-twisted scaffold and twisted scaffold [75].

**Table 9:** The braiding and twisting angles associated to each braided scaffold, braided-twisted scaffold and twisted scaffold. Reprinted with permission from [75]

Scaffold levels	Twisting angles^a^ (degrees)		
Fiber twisted to form fiber bundles	78 ± 3.4	69 ± 4.0	60 ± 4.5
Fiber bundles twisted to form yarns	83 ± 2.1	72 ± 2.3	62 ± 4.5
Yarns twisted to form scaffolds	79 ± 1.4	68 ± 3.8	62 ± 4.5
Scaffold	2 braid	4 braid	6 braid
Braiding angle (degrees)	78 ± 1.8	69 ± 2.7	61 ± 3.4

^a^The twisting angles are arranged into structures (fiber bundles, yarns and scaffolds)

**Fig. 19:**
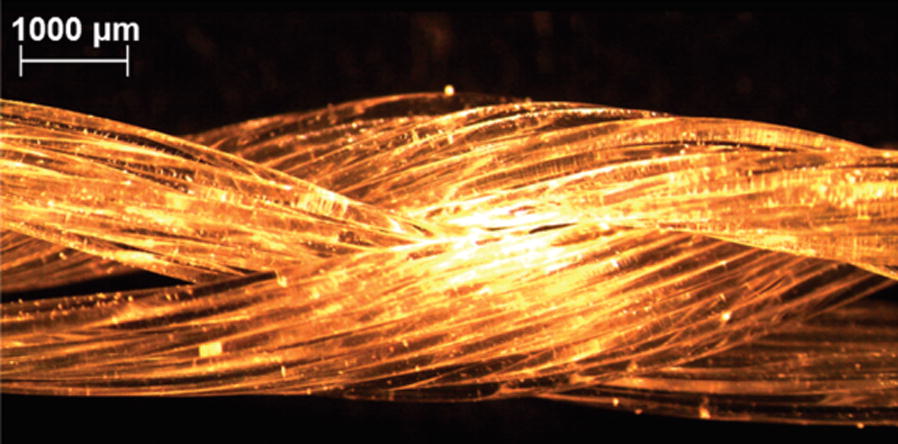
Optical microscopy picture of the ligament tissue engineering scaffold (Reprinted with permission from The Royal Society of Chemistry [123])

An optical microscopy of a braided-twisted scaffold for ligament regeneration developed by Leroy et al. [123], made of PLA combined with Pluronic or Tetronic (poly(ethylene oxide–propylene oxide co polymers), is illustrated in Fig. 19. Both types of scaffolds presented stress at failure compatible with that of ACL. Besides, in vitro tests with MSCs revealed cytocompatibility of both scaffolds, suggesting that the twisted-braided shape did not cause any significant loss of cell viability and enhanced cell proliferation [123].

Other common approaches for ligament/tendon regeneration combine fibrous or spongy scaffolds with gels of fibrin, Col or HA, for cell seeding, attempting to improve their biocompatibility, but these exhibited lack of mechanical properties and are unstable in a dynamic situation, such as in the knee joint [33, 71].

Coating of scaffolds with Col, HA or nanofibers, as well as the addition of growth factors, has been reported [80, 81, 103] as favoring cell attachment and proliferation, and ECM deposition [33]. The architecture of the scaffold can be modified in terms of pore diameter, porosity, surface area, by varying the fiber composition, diameter, braiding and twisting angles as well as yarn density [33].

**Fig. 20:**
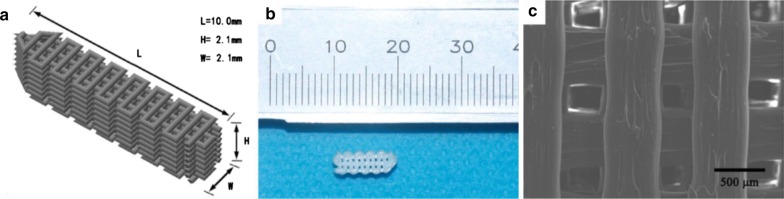
**a** 3D view of the theoretical designed PLA screw-like scaffold structure. **b** The prepared PLA screw-like scaffold. **c** The SEM image of the PLA scaffold surface with well-defined orthogonal structure (Reprinted with permission from [114])

Most of conventional methods used to produce TE scaffolds lack the ability to obtain highly repeatable designs with precise, well-defined micro- and nanoscale structures [124]. 3D-printing enables the production of scaffolds with patient-specific requirements [124] and it has recently been suggested for the production of screw-like scaffolds for tendon/ligament scaffolds [114, 125]. This kind of scaffold could fix the tendon/ligament graft, and provide adequate space for bone ingrowth around the graft [114]. 3D printing offers control over the architecture of the scaffold, such as porosity, thus controlling physical properties [126]. It follows a procedure based on the layer-by-layer deposition of the material, from bottom to top, to build a 3D product directly from a CAD model [127, 128]. 3D processes provide increased speed, customization and efficiency, not involving toxic solvents [127, 129, 130]. Figure 20 illustrates a 3D printed PLA screw-like scaffold developed by Liu et al. [114] for ligament applications, whose mechanical and biological properties were reported above.

Advances in 3D printing have increased feasibility towards the synthesis of living tissues-bioprinting [131]. This technology is based on a precise deposition of biomaterials, either encapsulating cells or loaded with cells later on, and growth factors, in micrometer scale to produce a bioidentical tissue [131, 132]. Several research groups have bioprinted materials and cells for musculoskeletal applications including bone, cartilage, muscle, tendon and ligament tissues. However, there are significant challenges to be resolved in terms of technological progresses [133, 134].

## Conclusions

Tissue engineering is a promising alternative approach to the current surgery procedures for tendon/ligament repair. Its goal is to provide a complete regeneration of the damaged tissue, recovering its native architecture and functionality. A wide variety of biodegradable polymers and composites has been proposed for that purpose. Col and PLLA are the most used materials to produce biodegradable scaffolds. Given the complex structure of native tissues, the production of fiber-based scaffolds has been the preferred option for tendon/ligament scaffolds. Despite the remarkable progress made in this field, the current TE approaches still present limitations in terms of mechanical properties, degradation rate and biological response that are necessary to overcome. In the future, new strategies such as 3D printing may provide a rapid and promising solution for the production of tendon/ligament scaffolds.

## Data Availability

Not applicable.

## Abbreviations

ACL: anterior cruciate ligament
FDA: Food and Drug Administration
LARS: Ligament Augmentation and Reconstruction System
TE: tissue engineering
ECM: extracellular matrix
ESCs: embryonic stem cells
iPSC: induced pluripotent stem cells
BMSCs: bone marrow-derived mesenchymal stem cells
AMSCs: adipocyte mesenchymal stem cells
QDs: quantum dots
MSCs: mesenchymal stem cells
IGF-I: insulin like growth factor I
TGF-β: transforming growth factor-β
bFGF: basic fibroblast growth factor
EU: European Union
EMA: European Medicines Agency
Col: collagen
CHI: chitosan
HA: hyaluronic acid
PLA: polylactic acid
PGA: polyglycolic acid
PLGA: poly(lactic-co-glycolic acid
PCL: poly(*ε*-caprolactone)
3D: three dimensional
PBS: phosphate buffered saline
PLLA: poly (l-lactic) acid
ALG: alginate
PT: patellar tendon
UTS: ultimate tensile strength
SEM: scanning electron microscopy
hFF: human foreskin fibroblasts
PLCL: poly(l-lactide-co-ε-caprolactone)
Max Load: maximum tensile load
MCL: medial collateral ligament
AT: Achilles tendon
Fn: fibronectin
PCLF: polycaprolactone fumarate
hTSCs: human tendon stem cells
NRS: aligned nanoyarns
CCK-8: cell counting kit-8
bFGF: basic fibroblast growth factor
CNT-COOH: COOH functionalized carbon nanotubes
GNP: graphene nanoplatelets
HDF: human dermal fibroblasts
Exp: experimental group
Ctrl: control group
w: week
NRAT: normal rabbit tendon
ATFs: Achilles tendon fibroblasts
PEGDA: polyethylene glycol diacrylate
PD: polydioxanone
SWNTs: single walled nanotubes)
P3HB: poly(3-hydroxybutyrate)
WJ-MSCs: Wharton’s jelly mesenchymal stem cells
CAD: computer-aided design
